# Supplementary material for: Microbial induced calcite precipitation can consolidate martian and lunar regolith simulants
Source: PLoS One. 2022 Apr 14;17(4):e0266415. doi: 10.1371/journal.pone.0266415 (PMC9009621; doi:10.1371/journal.pone.0266415)
Supplement: S1 Fig — (PDF) [file pone.0266415.s001.pdf]

Supplementary Information

for

Microbial induced calcite precipitation can consolidate martian and lunar regolith simulants

Rashmi Dikshit[1], Nitin Gupta[1], Arjun Dey[2], Koushik Viswanathan[1], Aloke Kumar[1]*

[1]Department of Mechanical Engineering, Indian Institute of Science, Bangalore 560012

[2] Thermal Systems Group, U. R. Rao Satellite Centre (Formerly ISRO Satellite Centre), Vimanapura Post, Bangalore, 560017, India

*Corresponding author: alokekumar@iisc.ac.in

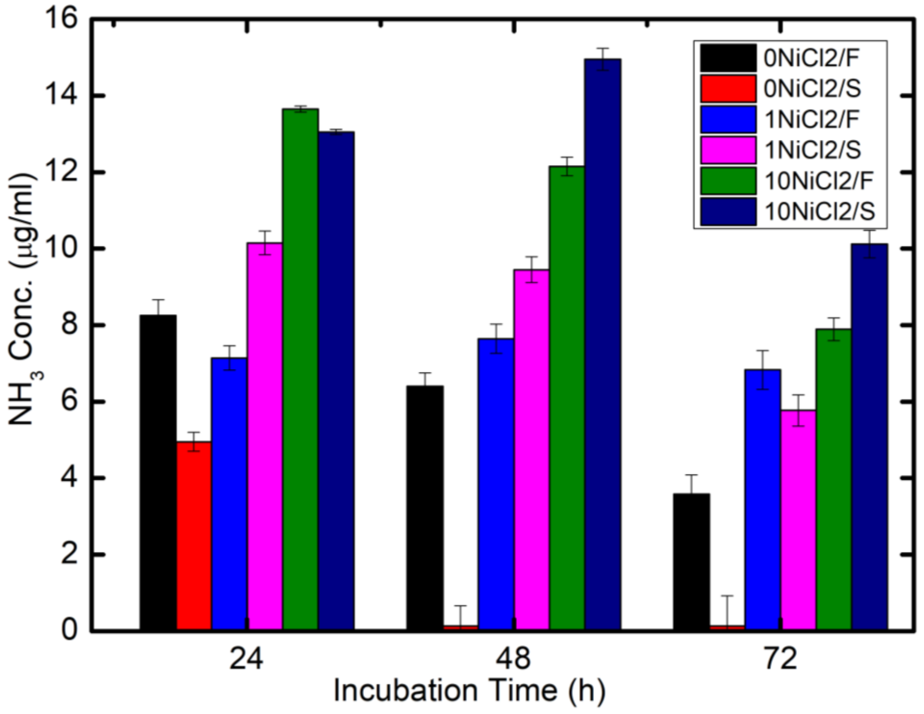

Fig. S1: Screening of temporal evolution of ammonium ion concentration with the supplementation of different concentration of $NiCl_2$ in flask condition and with soil separately (F denotes flask condition and S in the soil)
