# Supplementary material for: Microbial induced calcite precipitation can consolidate martian and lunar regolith simulants
Source: PLoS One. 2022 Apr 14;17(4):e0266415. doi: 10.1371/journal.pone.0266415 (PMC9009621; doi:10.1371/journal.pone.0266415)
Supplement: S1 Data — (ZIP) [file pone.0266415.s002.zip › Plos one_data file/raw_data_set/biochemical_assay_data.pdf]

Data recorded for biochemical assay pertaining to growth pattern with different reatments

pH variation for various treatments

Time (hr)	SM	standard error	SM-GG	standard error	SM-N	Error	SM-GG-N	standard error
0	6.5	0	6.5	0	6.5	0.012	6.5	0
3	6.6	0.05774	6.7	0.1	6.53	0.07321	6.53	0.05774
6	6.6	0.09018	6.7	0.03205	7	0.08166	6.9	0.05774
12	7.5	0.05292	6.64	0.05	8.1	0.09101	7.2	0.05774
18	8.1	0.05508	7.9	0.08544	8.9	0.08073	9.3	0.05
24	8.5	0.01	8.7	0.07211	9.1	0.08258	9.5	0.05774
36	8.84	0.05292	9.1	0.04619	9.2	0.07166	9.6	0.10066
48	8.8	0.08	9.5	0.06	9.2	0.09	9.7	0.089

OD (at 600 nm) variation for various treatments

Time (hr)	SM	standard error	SM-GG	standard error	SM-N	standard error	SM-GG-N	standard error
0	0.03433	0.08146	0.03633	0.03215	0.03633	0.02082	0.036	1E-3
3	0.05267	0.07258	0.03733	0.01528	0.041	1E-3	0.03467	0.07726
6	0.37167	0.08353	0.036	0	0.40667	0.07572	0.043	0.089
12	0.72667	0.09275	0.159	0.08544	0.85667	0.02517	0.36667	0.09215
18	1.19667	0.08583	0.698	0.07211	1.2	0.01	0.808	0.09658
24	1.52	0.0915	1.46333	0.03055	1.6	0.03512	1.64333	0.09528
36	1.6	0.092	2.15333	0.05033	1.7	0.02646	2.3	0.0735
48	0.89	0.0934	2.2	0.087	0.91	0.069	2.3	0.07

Ammonium ion concentration variation for various treatments

Time (hr)	SM	standard error	SM-GG	standard error	SM-N	standard error	SM-GG-N	standard error
0	0.08	0.9	0.09	0.78	0.1	0.89	0.11	0.82
3	0.1	0.12	0.15	0.57	0.11	0.8	0.12	0.912
6	1.26	0.36056	1.35	0.89	2.92	0.78	3.5	0.811
12	3.9	0.245	5.1	0.13	7.9	0.1	5.9	0.40415
18	6.5	0.35333	7.467	0.42333	10.9	0.5	12.8	0.7
24	8.8	0.75167	10.5	0.80667	15.9	0.5	17.8	0.5
36	10.23	0.70833	12.3	0.865	16.1	0.5	18.9	0.6773
48	11.34	0.73	17.78	0.91	16.2	0.7	19.98	0.68
