# Supplementary material for: Microbial induced calcite precipitation can consolidate martian and lunar regolith simulants
Source: PLoS One. 2022 Apr 14;17(4):e0266415. doi: 10.1371/journal.pone.0266415 (PMC9009621; doi:10.1371/journal.pone.0266415)
Supplement: S1 Data — (ZIP) [file pone.0266415.s002.zip › Plos one_data file/raw_data_set/GCMS_data_IISc.pdf]

Comment: This report has been generated by MS Facility Division of Biological Sciences IISc, For the research work carried by Dr. Rashmi Dikshit

Sample SM-GG

8.296

Name: α-Phellandrene

Formula: $C_{10}H_{16}$

MW: 136 Exact Mass: 136.1252 CAS#: 99-83-2 NIST#: 3305 ID#: 13142

DB: replib

Other DBs: TSCA, RTECS, EINECS, IRDB

[illegible]

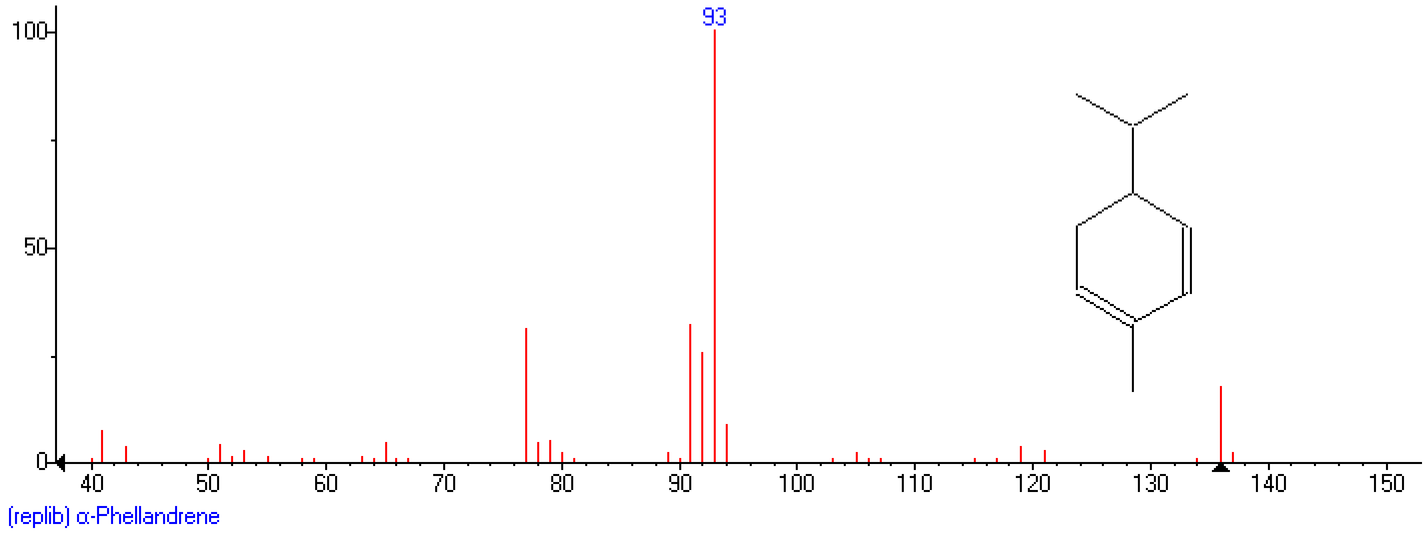

8.408

Name: 1,2,4-Benzenetricarboxylic acid, 1,2-dimethyl ester
Formula: $C_{11}H_{10}O_6$
MW: 238 Exact Mass: 238.047738 CAS#: 54699-35-3 NIST#: 47657
ID#: 166226 DB: mainlib
Other DBs: None
Contributor: CARL DJERASSI DEPT OF CHEM STANFORD UNIV

STANFORD CALIF 94305

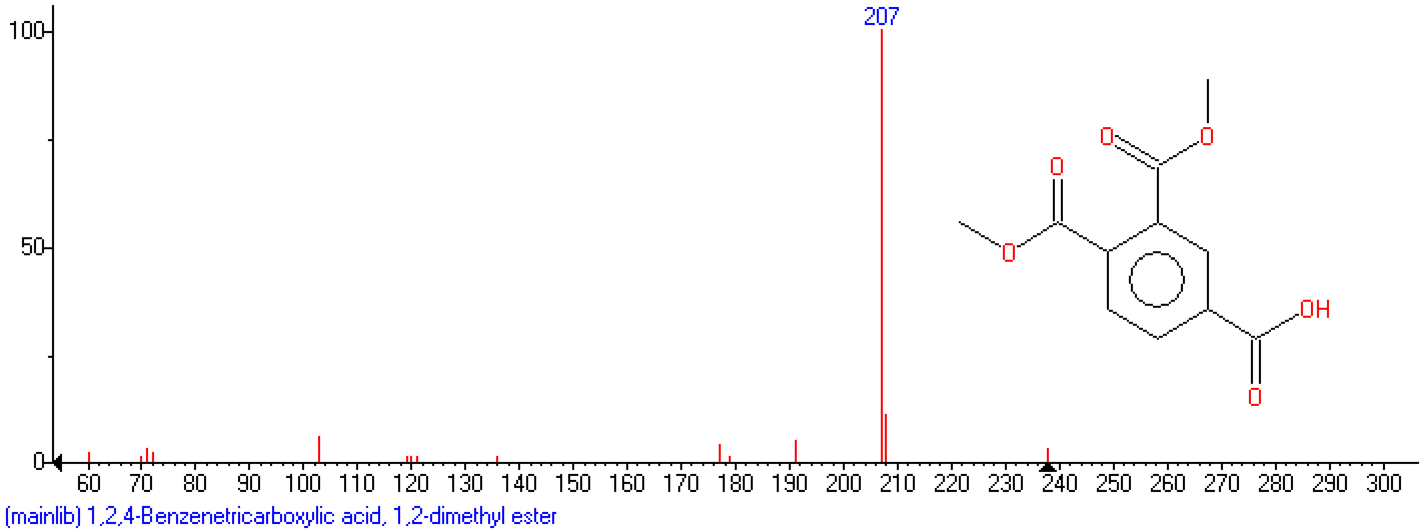

8.568

Name: Benzoic acid, 2-hydroxy-, 4-methylphenyl ester
Formula: $C_{14}H_{12}O_3$
MW: 228 Exact Mass: 228.078644 CAS#: 607-88-5 NIST#: 232781 ID#:

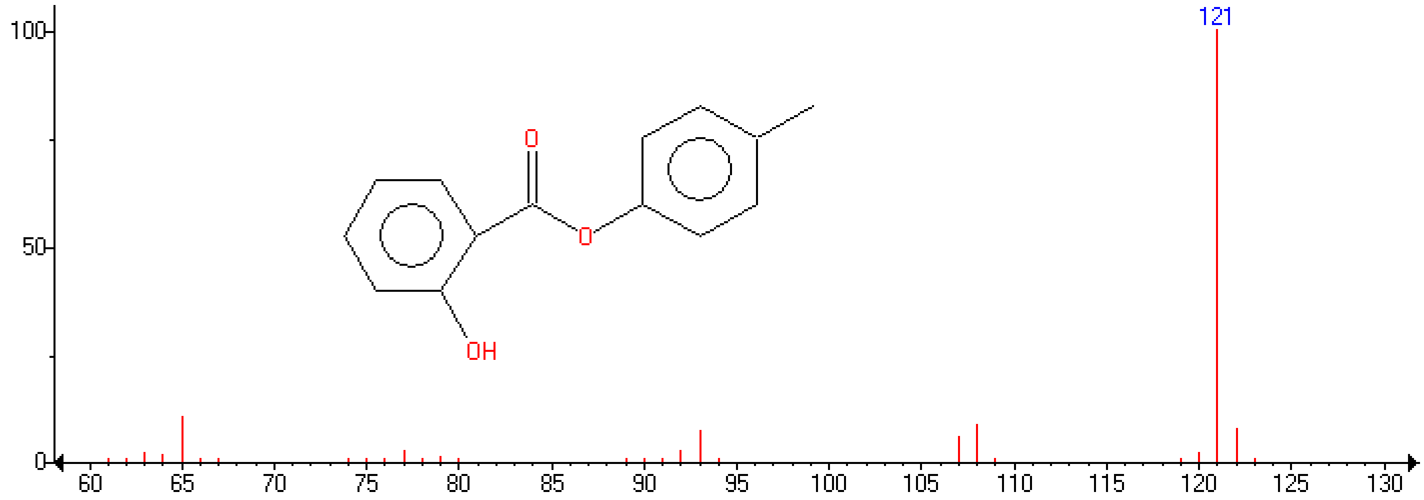

(mainlib) Benzoic acid, 2-hydroxy-, 4-methylphenyl ester

8.796

Name: Propanoic acid, 2-[(trimethylsilyl)oxy]-, trimethylsilyl ester
Formula: $C_9H_{22}O_3Si_2$

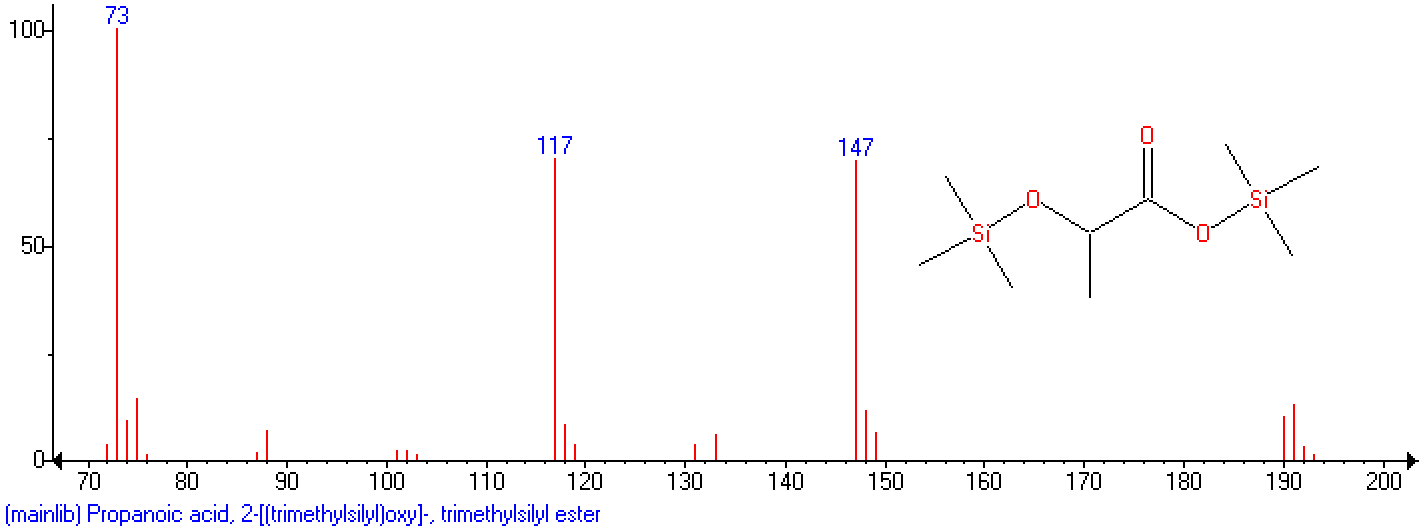

9.619

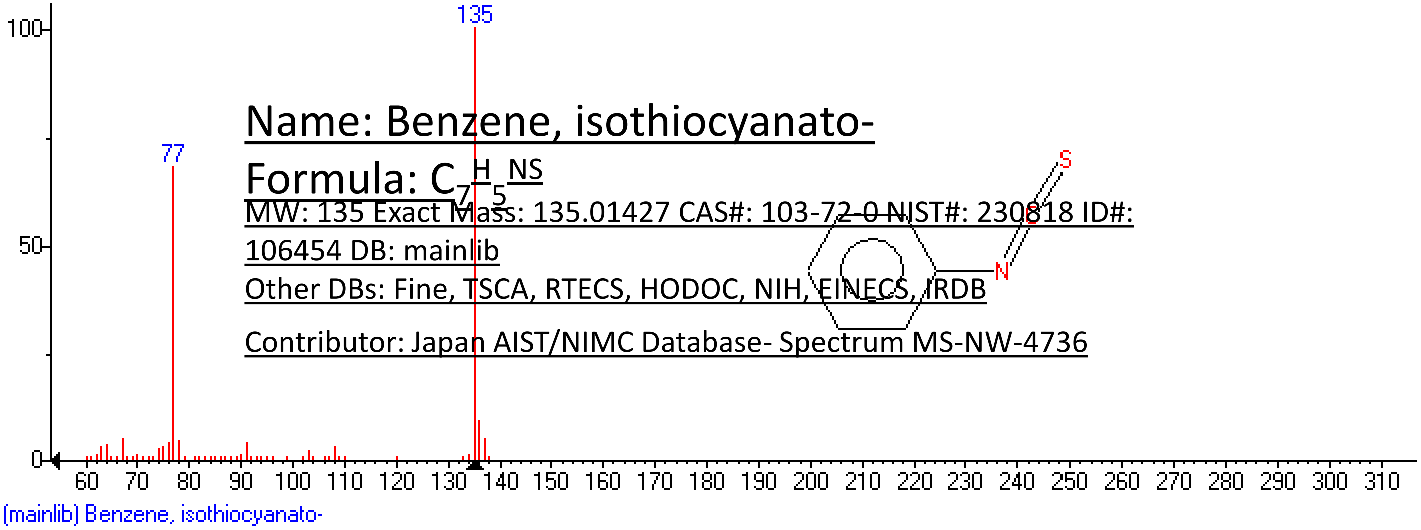

Name: Benzene, isothiocyanato-

Formula: C_7H_5NS

MW: 135 Exact Mass: 135.01427 CAS#: 103-72-0 NIST#: 230818 ID#: 106454 DB: mainlib

Other DBs: Fine, TSCA, RTECS, HODOC, NIH, EINECS, IRDB

Contributor: Japan AIST/NIMC Database- Spectrum MS-NW-4736

10.313

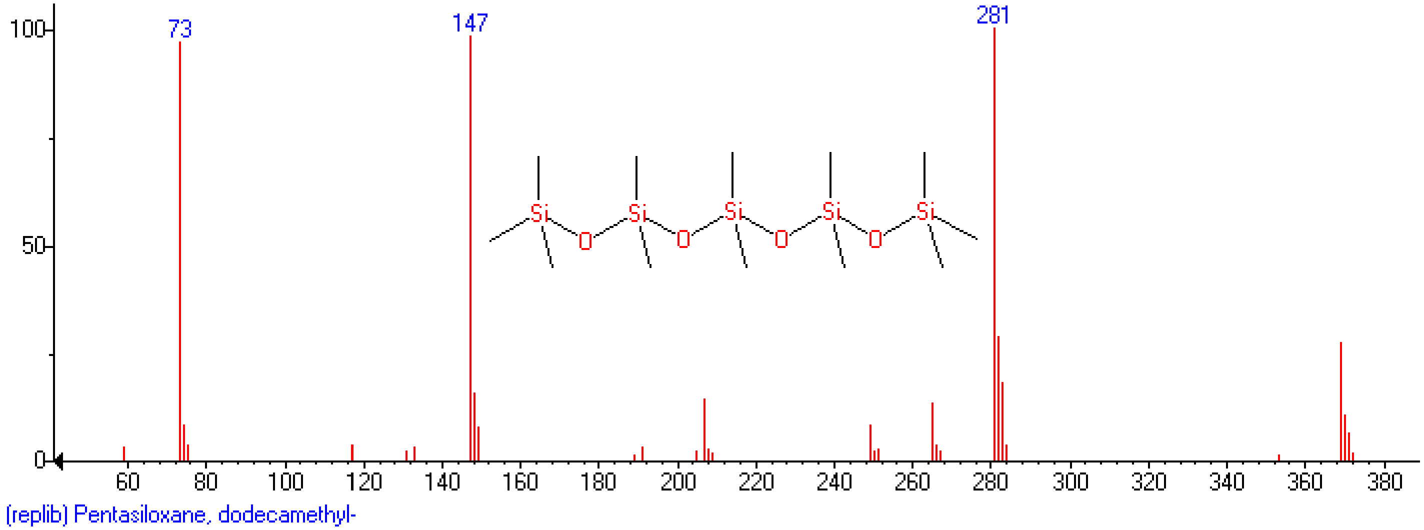

100
50
0
73
147
281
Si
O
Si
O
Si
O
Si
O
Si
60
80
100
120
140
160
180
200
220
240
260
280
300
320
340
360
380
(replib) Pentasiloxane, dodecamethyl-

10.813

Name: Cyclopropane, nonyl-

Formula: $C_{12}H_{24}$

MW: 168 Exact Mass: 168.1878 CAS#: 74663-85-7 NIST#: 62608 ID#: 18566 DB: mainlib

Other DBs: None

Contributor: D.HENNEBERG, MAX-PLANCK INSTITUTE, MULHEIM, WEST GERMANY

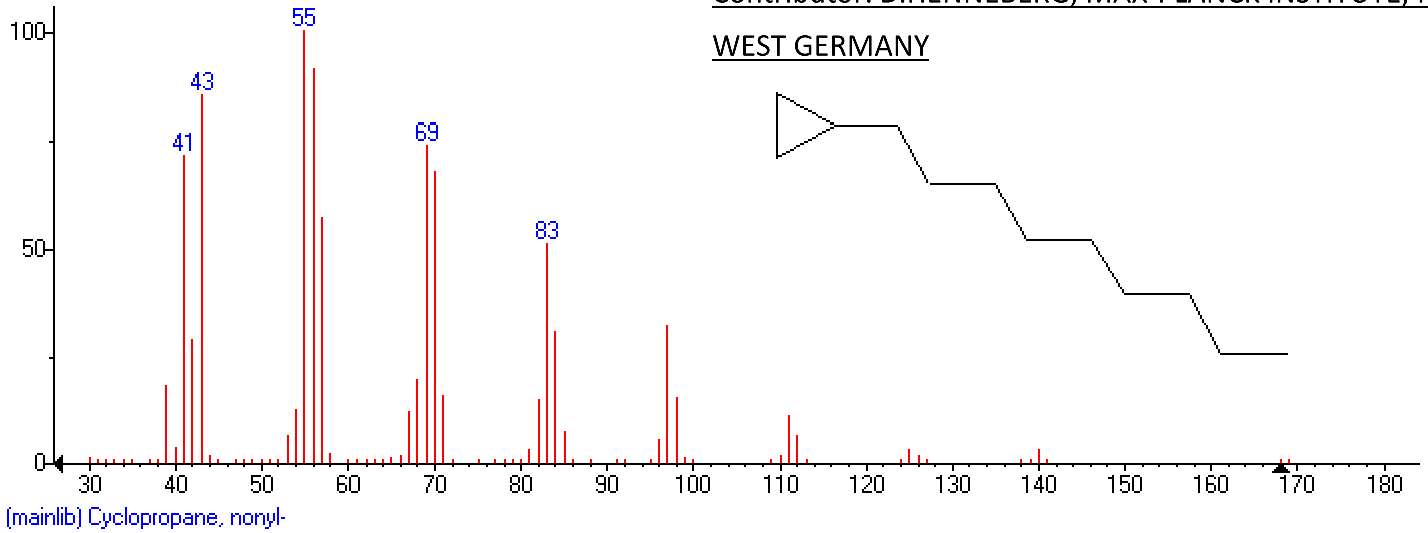

10.945

Name: Naphthalene

Formula: $C_{10}H_8$

MW: 128 Exact Mass: 128.0626 CAS#: 91-20-3 NIST#: 379701 ID#: 19026 DB: replib

Other DBs: Fine, TSCA, RTECS, EPA, HODOC, NIH, EINECS, IRDB

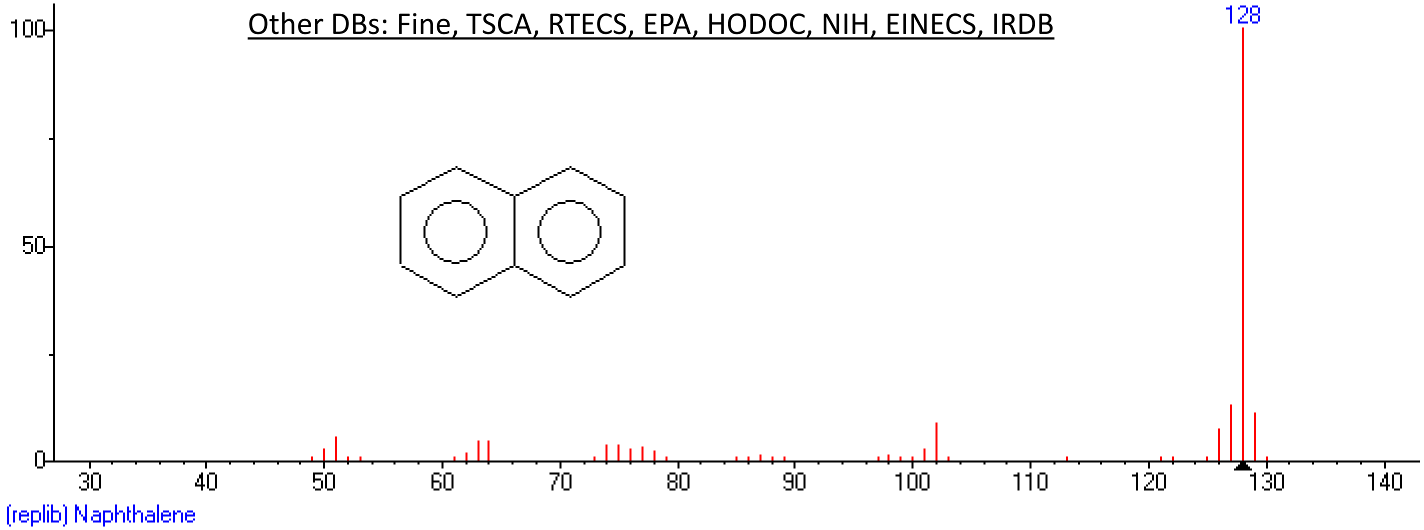

10.983

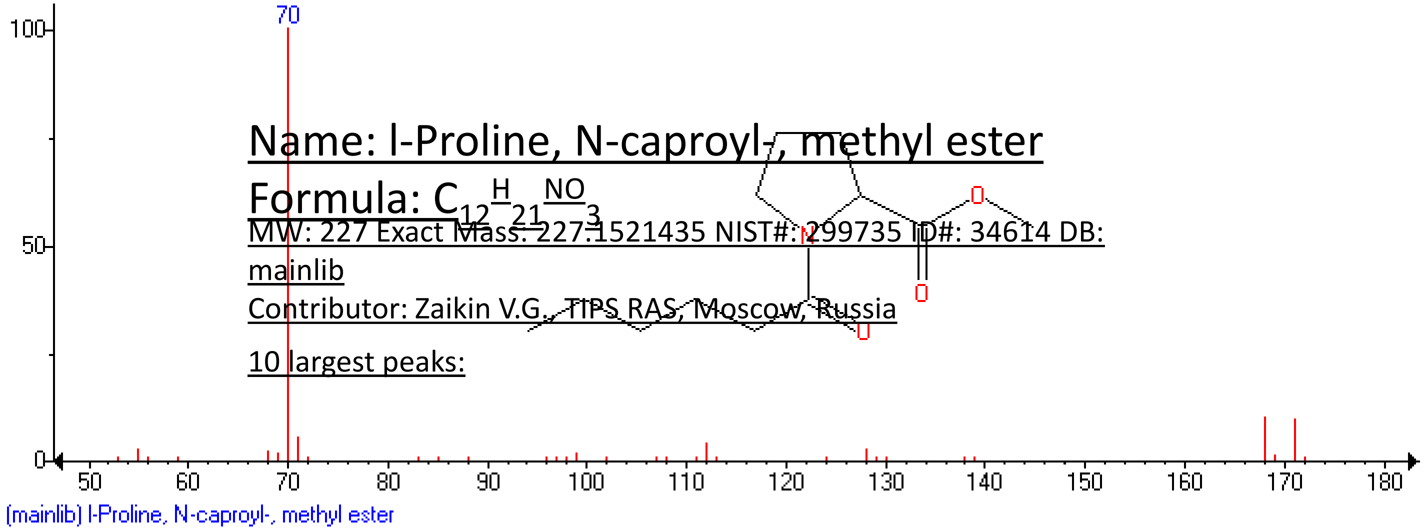

Name: l-Proline, N-caproyl-, methyl ester

Formula: $C_{12}H_{21}NO_3$

MW: 227 Exact Mass: 227.1521435 NIST#: 299735 ID#: 34614 DB: mainlib

Contributor: Zaikin V.G., TIPS RAS, Moscow, Russia

10 largest peaks:

11.058

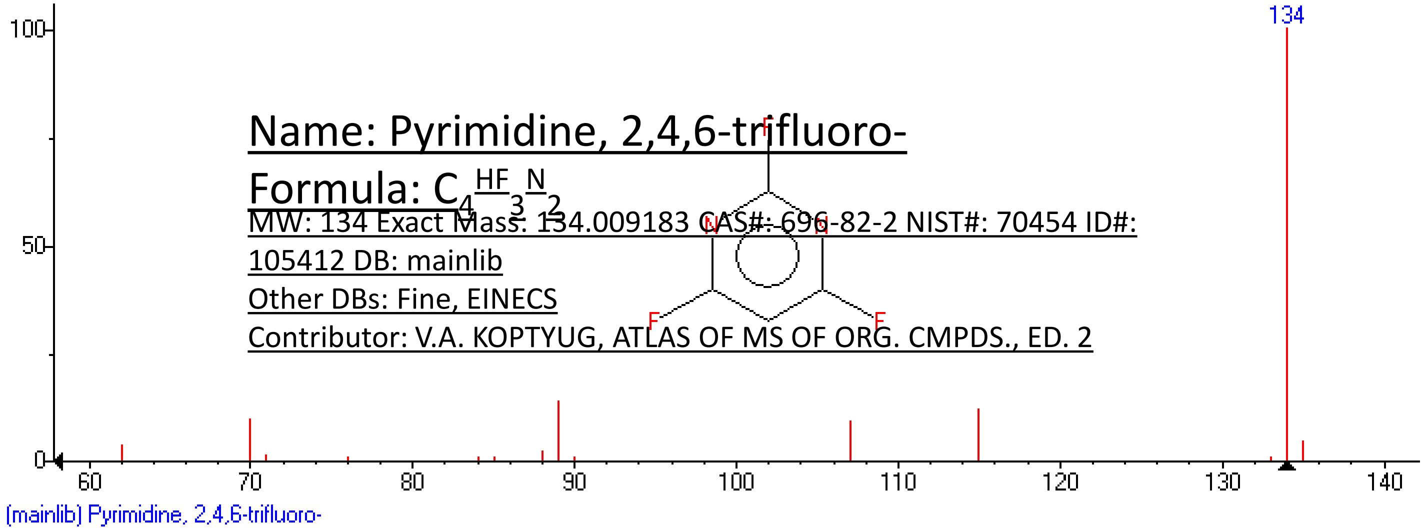

Name: Pyrimidine, 2,4,6-trifluoro-
Formula: $C_4HF_3N_2$
MW: 134 Exact Mass: 134.009183 CAS#: 696-82-2 NIST#: 70454 ID#: 105412 DB: mainlib
Other DBs: Fine, EINECS
Contributor: V.A. KOPTYUG, ATLAS OF MS OF ORG. CMPDS., ED. 2
F
N
N
F
F
134
100
50
0
60
70
80
90
100
110
120
130
140
(mainlib) Pyrimidine, 2,4,6-trifluoro-

12.194

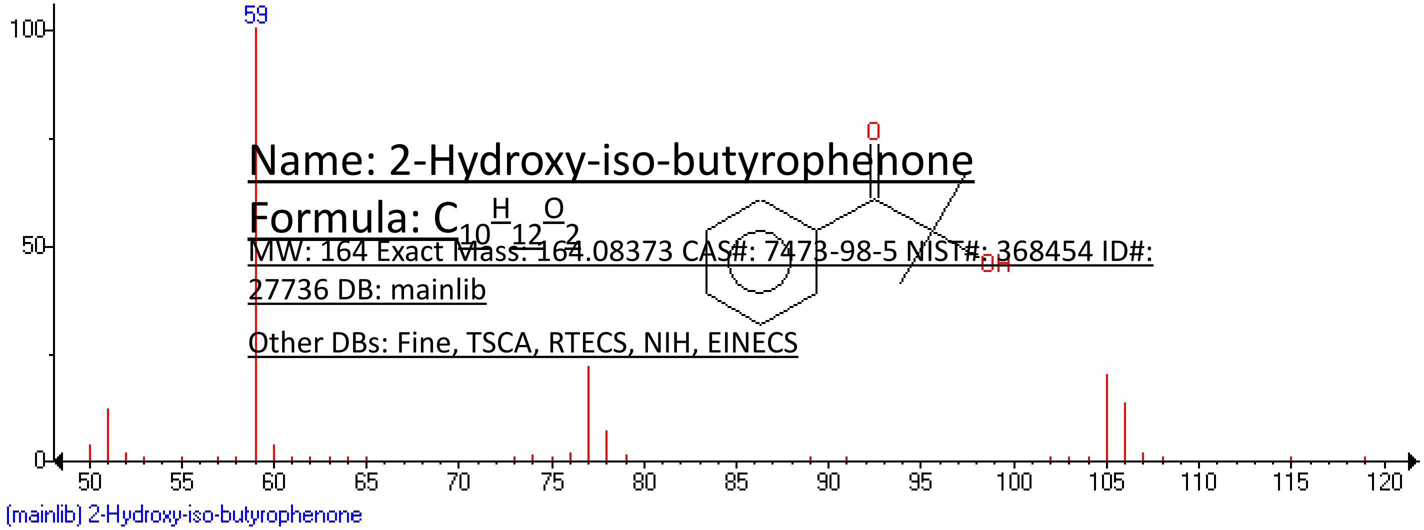

Name: 2-Hydroxy-iso-butyrophenone

Formula: $C_{10}H_{12}O_2$

MW: 164 Exact Mass: 164.08373 CAS#: 7473-98-5 NIST#: 368454 ID#: 27736 DB: mainlib

Other DBs: Fine, TSCA, RTECS, NIH, EINECS

12.238

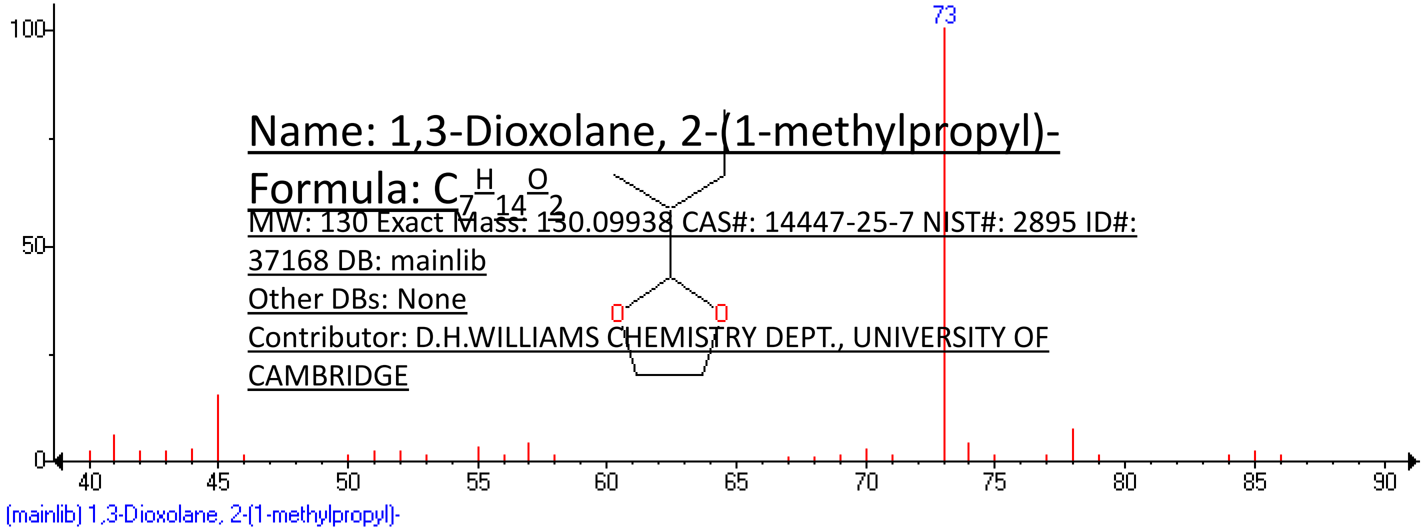

Name: 1,3-Dioxolane, 2-(1-methylpropyl)-

Formula: $C_7H_{14}O_2$

MW: 130 Exact Mass: 130.09938 CAS#: 14447-25-7 NIST#: 2895 ID#: 37168 DB: mainlib

Other DBs: None

Contributor: D.H.WILLIAMS CHEMISTRY DEPT., UNIVERSITY OF CAMBRIDGE

12.371

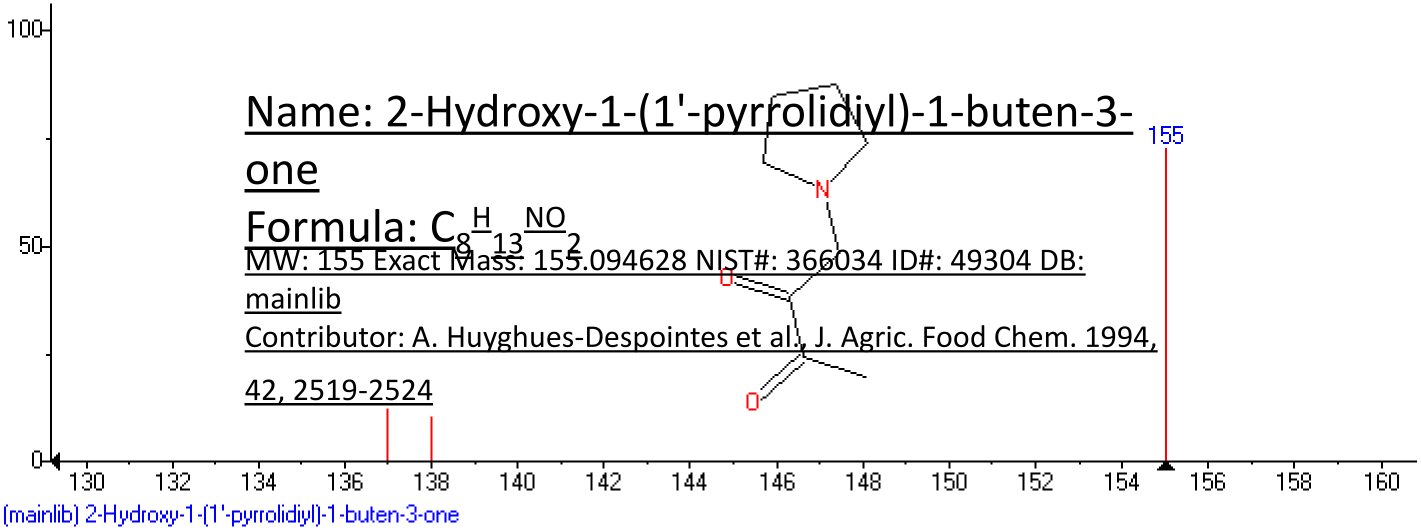

Name: 2-Hydroxy-1-(1'-pyrrolidiyl)-1-buten-3-one

Formula: $C_8H_{13}NO_2$

MW: 155 Exact Mass: 155.094628 NIST#: 366034 ID#: 49304 DB: mainlib

Contributor: A. Huyghues-Despointes et al., J. Agric. Food Chem. 1994, 42, 2519-2524

Name: 5-Octadecene, (E)-

13.548

Formula: $C_{18}H_{36}$

MW: 252 Exact Mass: 252.281701 CAS#: 7206-21-5 NIST#: 62810 ID#: 18641 DB: mainlib

Other DBs: None

Contributor: D.HENNEBERG, MAX-PLANCK INSTITUTE, MULHEIM, WEST GERMANY

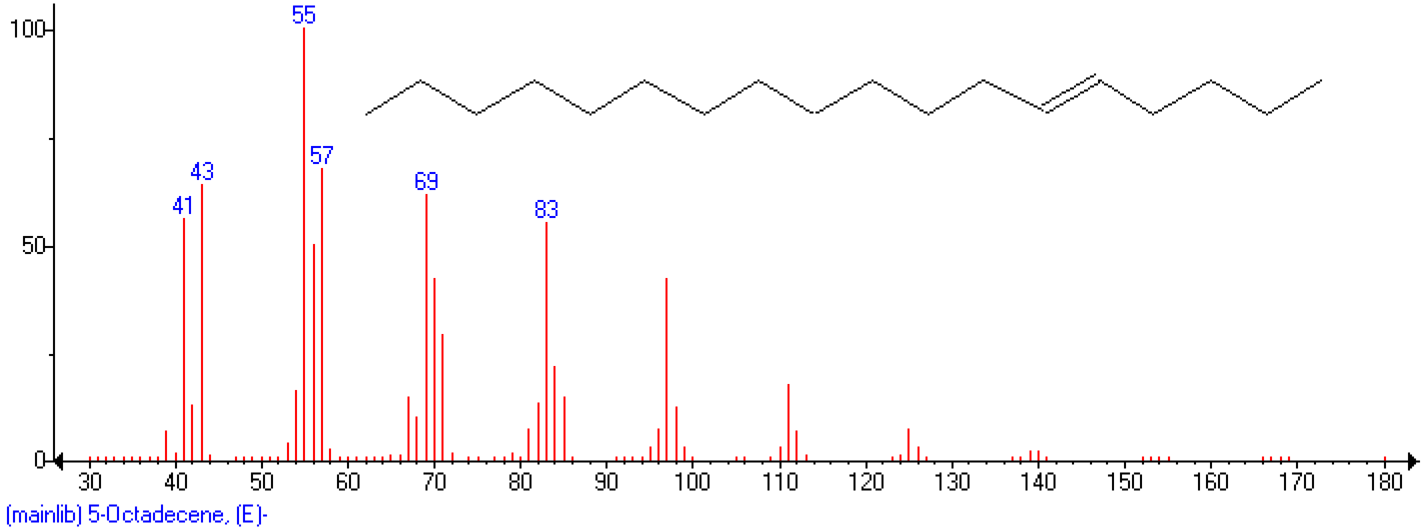

13.565

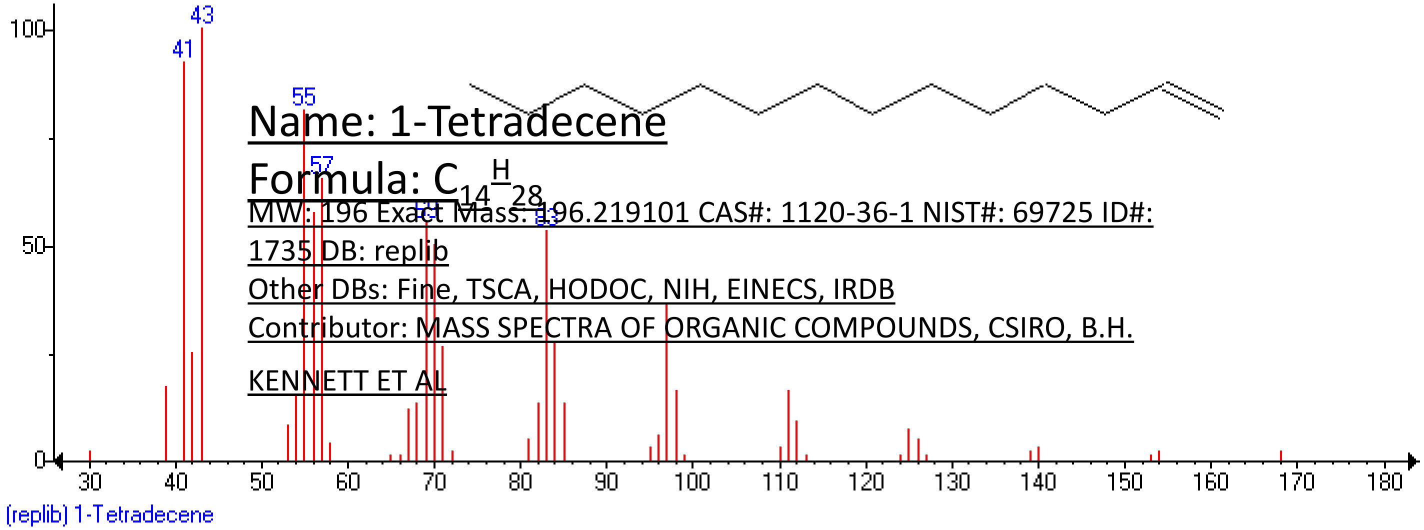

Name: 1-Tetradecene

Formula: $C_{14}H_{28}$

MW: 196 Exact Mass: 196.219101 CAS#: 1120-36-1 NIST#: 69725 ID#: 1735 DB: replib

Other DBs: Fine, TSCA, HODOC, NIH, EINECS, IRDB

Contributor: MASS SPECTRA OF ORGANIC COMPOUNDS, CSIRO, B.H. KENNETT ET AL

13.704

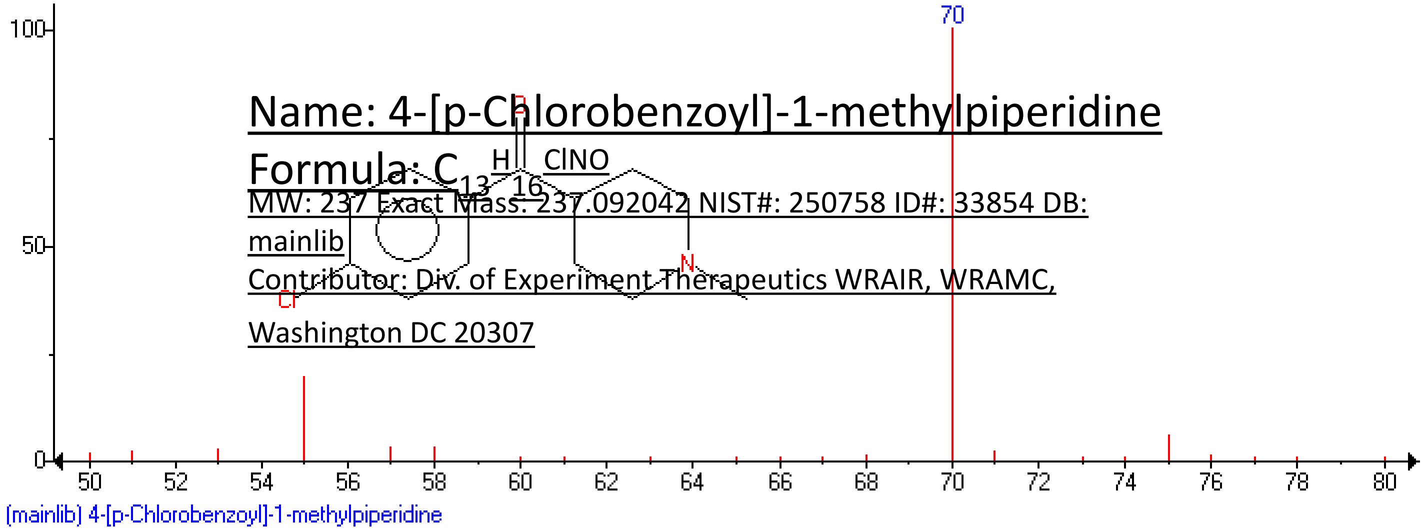

Name: 4-[p-Chlorobenzoyl]-1-methylpiperidine

Formula: $C_{13}H_{16}ClNO$

MW: 237 Exact Mass: 237.092042 NIST#: 250758 ID#: 33854 DB: mainlib

Contributor: Div. of Experiment Therapeutics WRAIR, WRAMC, Washington DC 20307

13.721

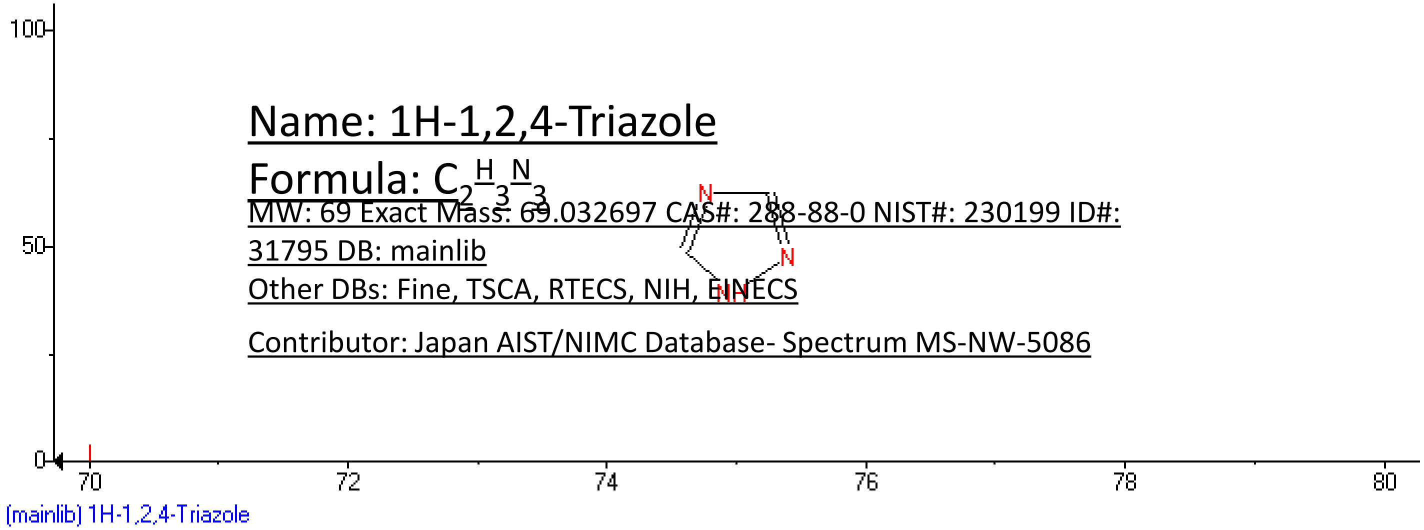

Name: 1H-1,2,4-Triazole

Formula: $C_2H_3N_3$

MW: 69 Exact Mass: 69.032697 CAS#: 288-88-0 NIST#: 230199 ID#: 31795 DB: mainlib

Other DBs: Fine, TSCA, RTECS, NIH, EINECS

Contributor: Japan AIST/NIMC Database- Spectrum MS-NW-5086

14.418

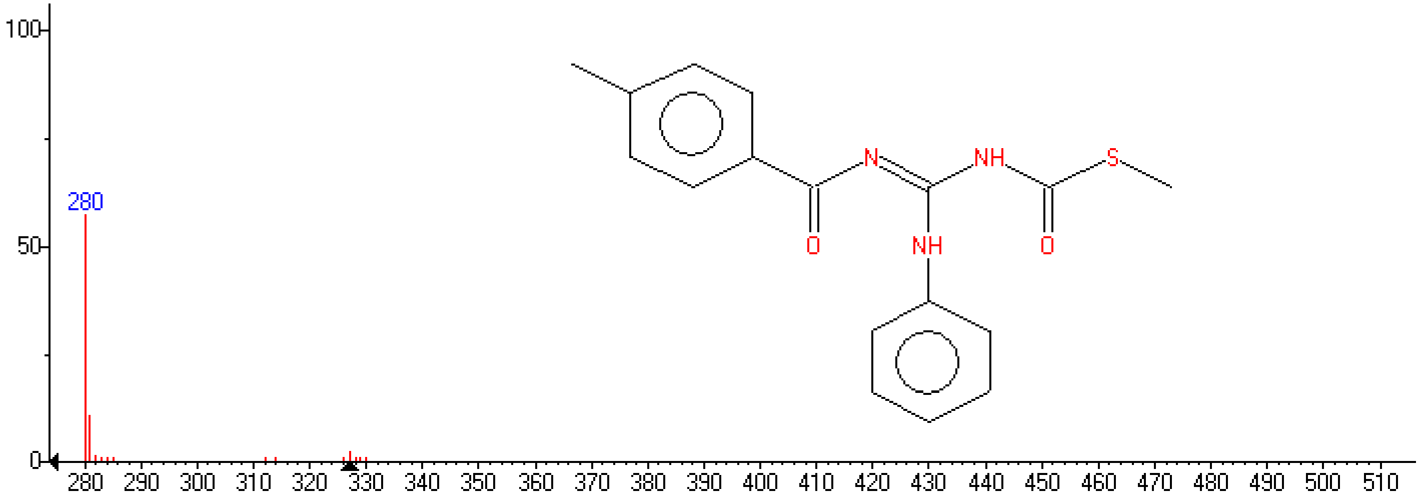

(mainlib) Carbamothioic acid, [[(4-methylbenzoyl)amino](phenylamino)methylene]-, S-methyl ester

Name: Carbamothioic acid, [[(4-methylbenzoyl)amino](phenylamino)methylene]-, S-methyl ester

Formula: $C_{17}H_{17}N_3O_2S$

MW: 327 Exact Mass: 327.104147 CAS#: 79340-27-5 NIST#: 150206

ID#: 90162 DB: mainlib

Other DBs: None

Contributor: Chemical Concepts

15.014

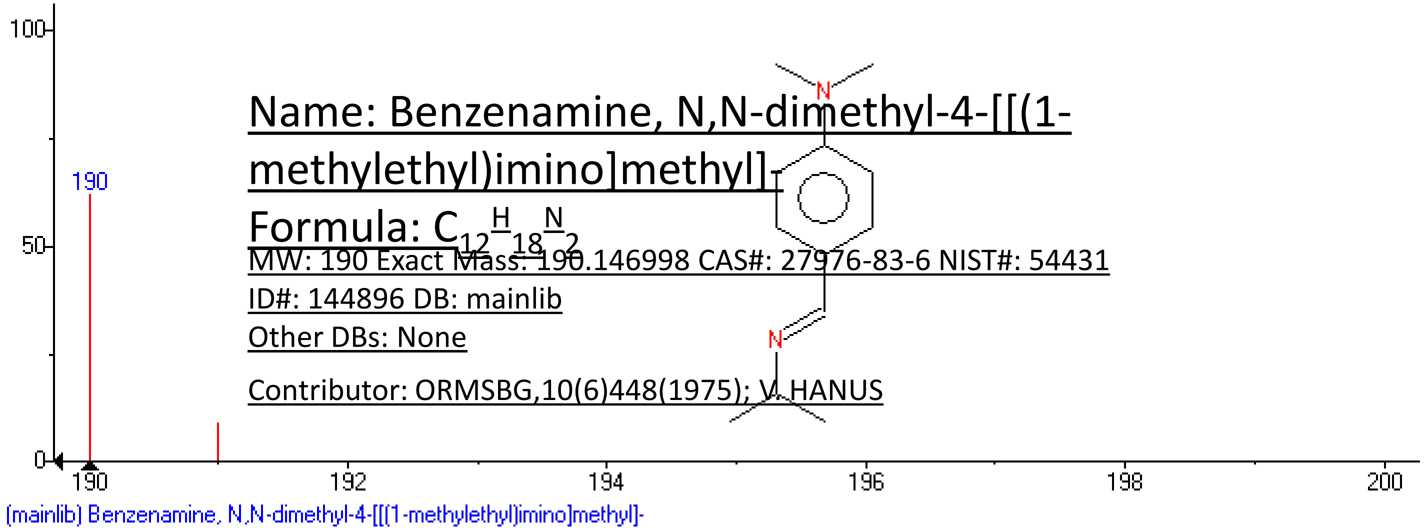

Name: Benzenamine, N,N-dimethyl-4-[[(1-methylethyl)imino]methyl]-
Formula: $C_{12}H_{18}N_2$
MW: 190 Exact Mass: 190.146998 CAS#: 27976-83-6 NIST#: 54431
ID#: 144896 DB: mainlib
Other DBs: None
Contributor: ORMSBG,10(6)448(1975); V. HANUS
100
50
0
190
190
192
194
196
198
200
N
N
(mainlib) Benzenamine, N,N-dimethyl-4-[[(1-methylethyl)imino]methyl]-

16.031

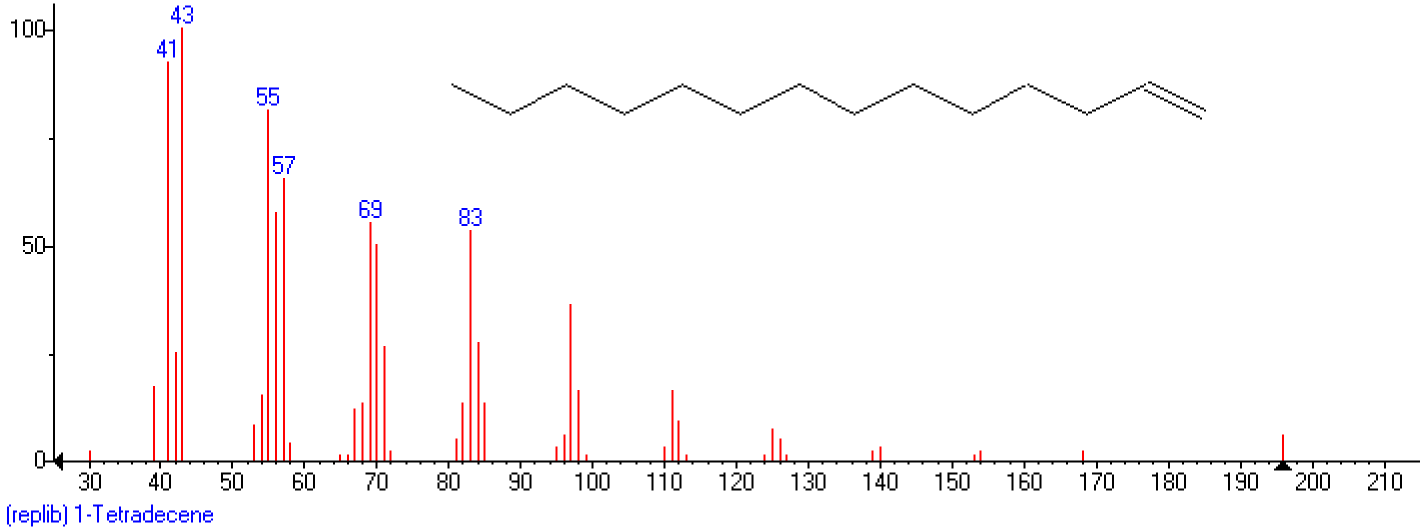

Name: 1-Tetradecene

Formula: $C_{14}H_{28}$

MW: 196 Exact Mass: 196.219101 CAS#: 1120-36-1 NIST#: 69725 ID#: 1735 DB: replib

Other DBs: Fine, TSCA, HODOC, NIH, EINECS, IRDB

Contributor: MASS SPECTRA OF ORGANIC COMPOUNDS, CSIRO, B.H. KENNETT ET AL

18.058

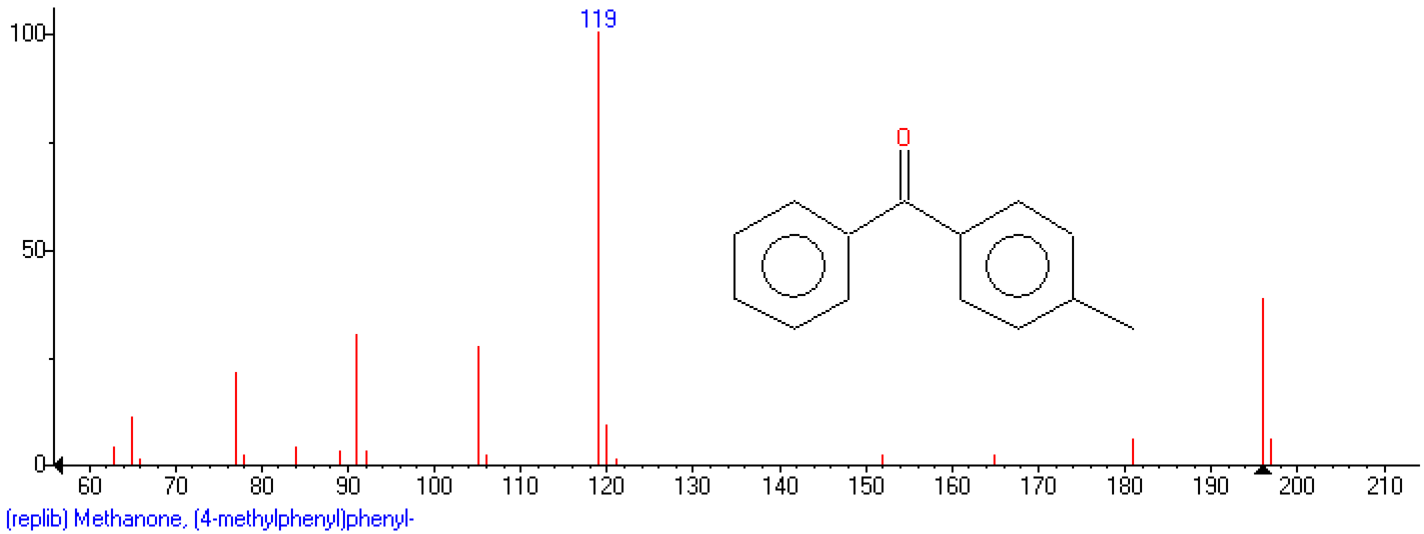

Name: Methanone, (4-methylphenyl)phenyl-

Formula: $C_{14}H_{12}O$

MW: 196 Exact Mass: 196.088815 CAS#: 134-84-9 NIST#: 22409 ID#: 17406 DB: replib

Other DBs: Fine, TSCA, RTECS, HODOC, NIH, EINECS

10 largest peaks:

18.248

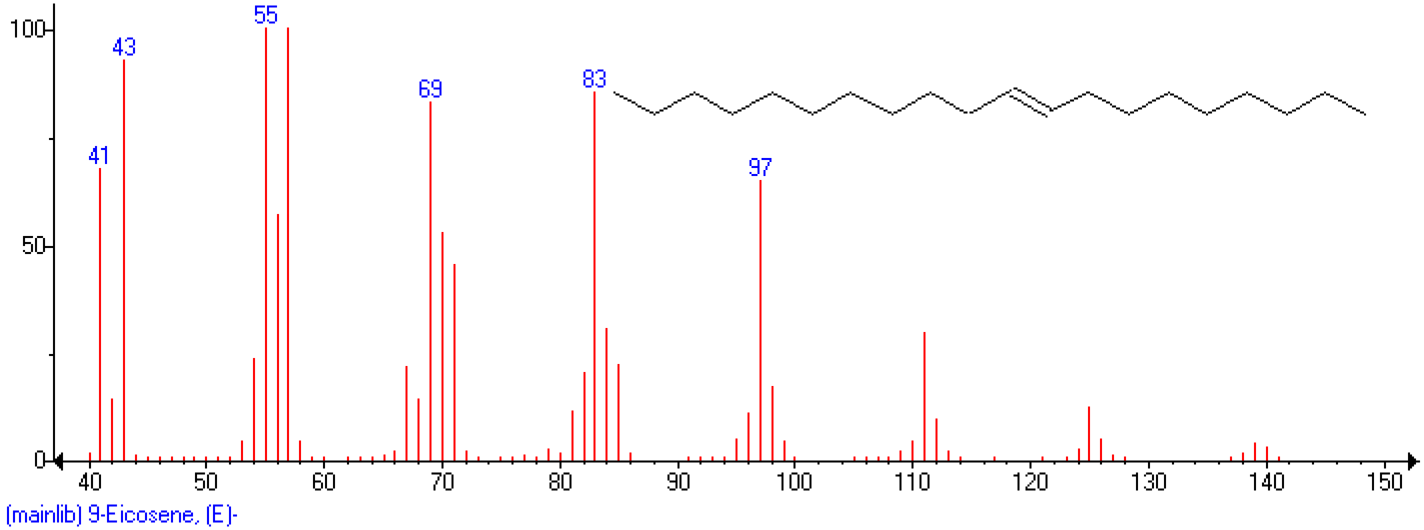

Name: 9-Eicosene, (E)-

Formula: $C_{20}H_{40}$

MW: 280 Exact Mass: 280.313002 CAS#: 74685-29-3 NIST#: 62815

ID#: 23002 DB: mainlib

Other DBs: None

Contributor: D.HENNEBERG, MAX-PLANCK INSTITUTE, MULHEIM, WEST GERMANY

10 largest peaks:

20.265

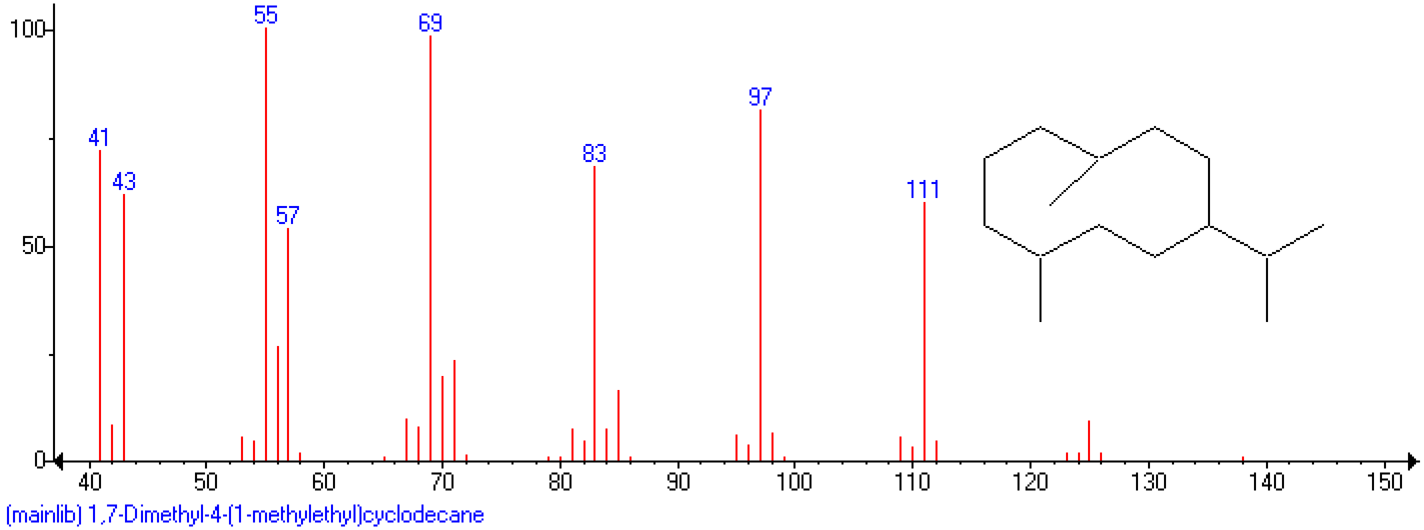

Name: 1,7-Dimethyl-4-(1-methylethyl)cyclodecane

Formula: $C_{15}H_{30}$

MW: 210 Exact Mass: 210.234751 CAS#: 645-10-3 NIST#: 249054 ID#: 18985 DB: mainlib

Other DBs: None

Contributor: TNO Volatile Compounds in Food - Chemical Concepts

21.085

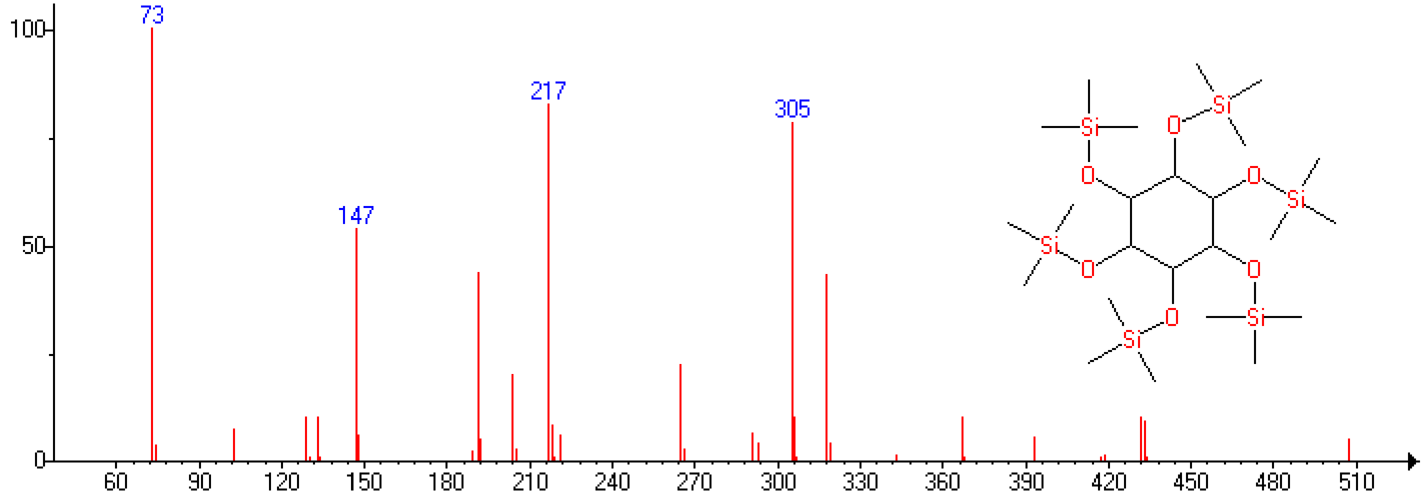

[replib] Myo-Inositol, 1,2,3,4,5,6-hexakis-O-(trimethylsilyl)-

Name: Myo-Inositol, 1,2,3,4,5,6-hexakis-O-(trimethylsilyl)-

Formula: $C_{24}H_{60}O_{6}Si_{6}$

MW: 612 Exact Mass: 612.30055 CAS#: 2582-79-8 NIST#: 18521 ID#: 9532 DB: replib

Other DBs: None

10 largest peaks:

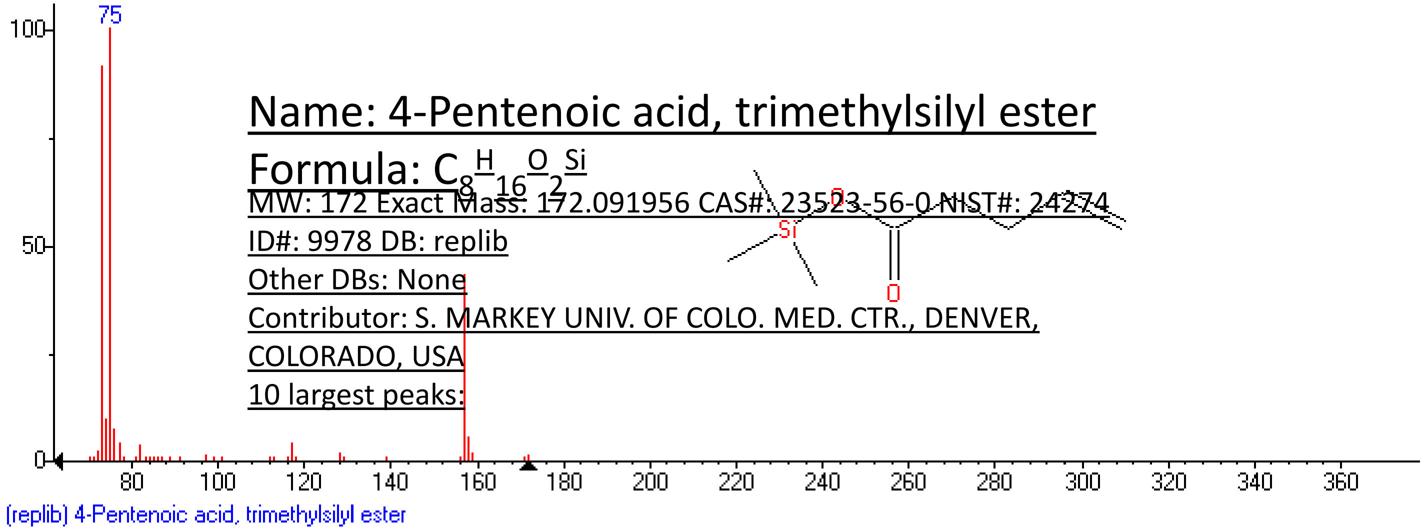

Name: 4-Pentenoic acid, trimethylsilyl ester

Formula: $C_8H_{16}O_2Si$

MW: 172 Exact Mass: 172.091956 CAS#: 23523-56-0 NIST#: 24274

ID#: 9978 DB: replib

Other DBs: None

Contributor: S. MARKEY UNIV. OF COLO. MED. CTR., DENVER, COLORADO, USA

10 largest peaks:

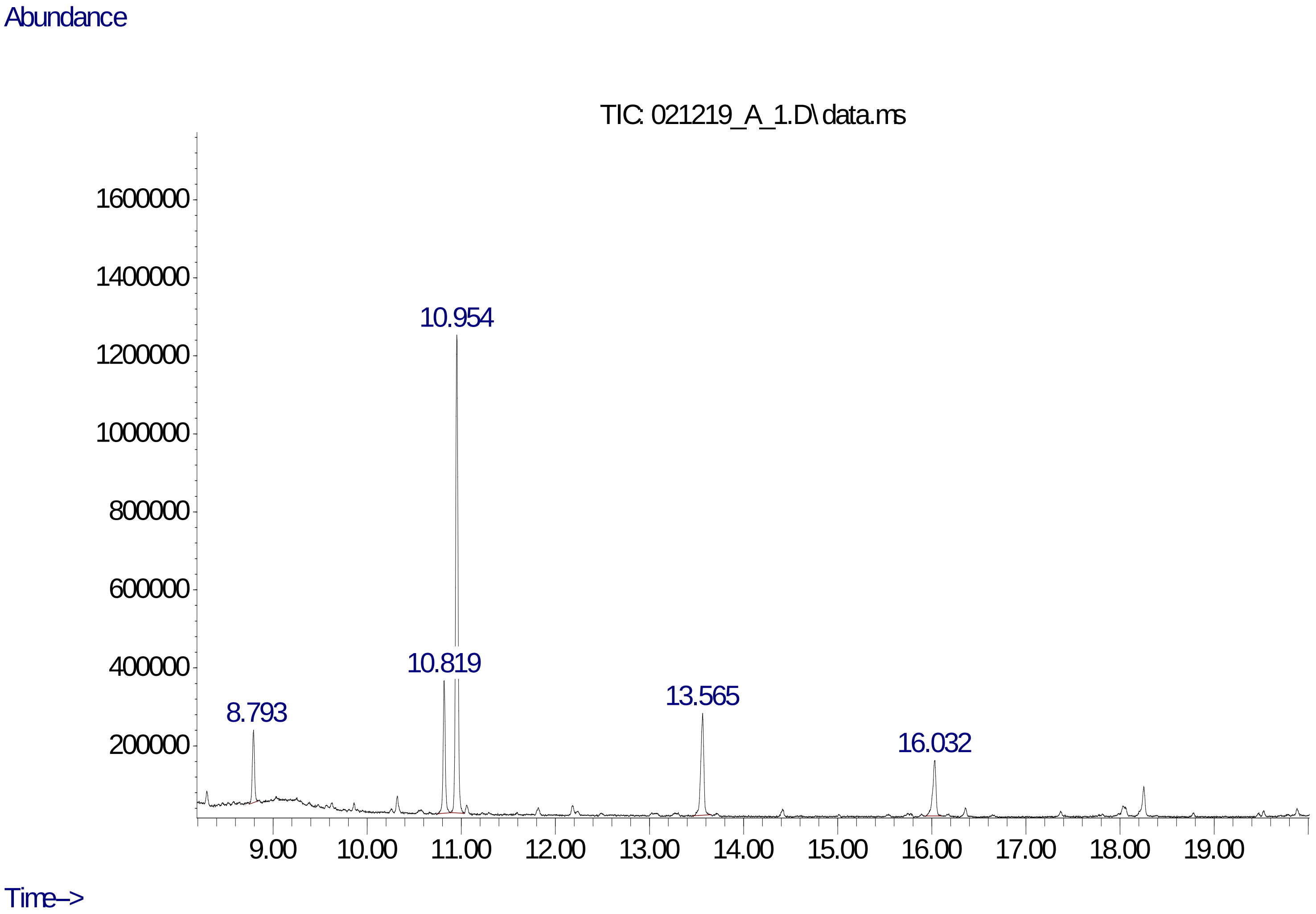
Abundance
TIC: 021219_A_1.D\data.ms
1600000
1400000
1200000
1000000
800000
600000
400000
200000
10.954
10.819
8.793
13.565
16.032
9.00
10.00
11.00
12.00
13.00
14.00
15.00
16.00
17.00
18.00
19.00
Time-->

- Data Path : G:\GC-MS data\IISc\2019\Rashmi\
- Data File : 021219_A_1.D
- Acq On : 29 Feb 2020 19:12 (#1); 29 Feb 2020 19:04 (#2)
- Operator : Muralidhar
- Sample : 021219_A_1
- Misc :
- ALS Vial : 2 Sample Multiplier: 1

- Integration Parameters: autoint1.e
- Integrator: ChemStation

- Method : D:\GCMS\IISc\2016\BC\CJB\Hex July yogi\130716_SA1.D\2016_KAMALRAJ_SPLIT25.M
- Title :

- Signal : TIC: 021219_A_1.D\data.ms

peak #	R.T. min	first scan	max scan	last scan	PK TY	peak height	corr. area	corr. % max.	% of total
1	8.793	165	178	189	BB 3	184744	2940595	13.56%	7.429%
2	10.819	753	774	795	BB	340314	5471475	25.23%	13.824%
3	10.954	798	814	836	BV	1227483	21684820	100.00%	54.787%
4	13.565	1550	1581	1605	BB 4	258835	5982176	27.59%	15.114%
5	16.032	2278	2306	2327	BB 4	142529	3501412	16.15%	8.846%

- Sum of corrected areas: 39580479
- Signal : 021219_A_1.D\FID1A.ch

peak #	R.T. min	Start min	End min	PK TY	peak height	corr. area	corr. % max.	% of total
1	2.996	2.288	3.082	BV	50843741	17019070020	46.05%	30.244%
2	4.187	3.082	4.295	VB	61695646	36961671773	100.00%	65.683%
3	5.399	5.095	5.672	BB	16240851	2291669783	6.20%	4.072%

- Sum of corrected areas: 56272411577

- 2016_KAMALRAJ_SPLIT25.M Fri Dec 06 15:45:43 2019

8.793

- Name: Propanoic acid, 2-[(trimethylsilyl)oxy]-, trimethylsilyl ester
- Formula: $C_9H_{22}O_3Si_2$
- MW: 234 Exact Mass: 234.110747 CAS#: 17596-96-2 NIST#: 158690 ID#: 9343 DB: replib
- Other DBs: None
- Contributor: Chemical Concepts
- 10 largest peaks:
- 73 999 | 147 871 | 117 824 | 191 245 | 45 239 |
- 190 189 | 148 146 | 75 135 | 66 124 | 43 110 |
- Synonyms:
- 1.Propionic acid, 2-(trimethylsiloxy)-, trimethylsilyl ester
- 2.Bis(trimethylsilyl)lactate
- 3.Lactic acid, bis(trimethylsilyl)oxy-, ester
- 4.2-Trimethylsilyloxypropionic acid,trimethylsilyl ester
- 5.Trimethylsilyl 2-[(trimethylsilyl)oxy]propanoate
- 6.Lactic acid, bis-TMS
- 7.Lactic acid, di-TMS
- 8.Lactic acid, O,O-TMS
- 9.Lactic acid, (2TMS)

- Estimated non-polar retention index (n-alkane scale):
- Value: 915 iu
- Confidence interval (Diverse functional groups): 89(50%) 382(95%) iu

10.819

- Name: 1-Dodecene
- Formula: $C_{12}H_{24}$
- MW: 168 Exact Mass: 168.1878 CAS#: 112-41-4 NIST#: 61826 ID#: 2092 DB: replib
- Other DBs: Fine, TSCA, HODOC, NIH, EINECS, IRDB
- Contributor: D.HENNEBERG, MAX-PLANCK INSTITUTE, MULHEIM, WEST GERMANY
- 10 largest peaks:
- 43 999 | 56 829 | 55 826 | 41 749 | 69 620 |
- 70 577 | 57 571 | 83 467 | 29 382 | 84 327 |
- Synonyms:
- 1.α-Dodecene
- 2.n-Dodec-1-ene
- 3.Adacene 12
- 4.Dodec-1-ene
- 5.α-Dodecylene
- 6.Dodecylene α-
- 7.Dodecene-1
- 8.Neodene 12
- 9.NSC 12016

- Estimated non-polar retention index (n-alkane scale):
- Value: 1204 iu
- Confidence interval (Hydrocarbons): 39(50%) 167(95%) iu

10.954

- Name: Naphthalene
- Formula: $C_{10}H_8$
- MW: 128 Exact Mass: 128.0626 CAS#: 91-20-3 NIST#: 379701 ID#: 19026 DB: replib
- Other DBs: Fine, TSCA, RTECS, EPA, HODOC, NIH, EINECS, IRDB
- Contributor: Drug Lab
- Related CAS#: 72931-45-4
- 10 largest peaks:
- 128 999 | 127 126 | 129 108 | 102 85 | 126 72 |
- 51 54 | 63 45 | 64 45 | 75 36 | 74 34 |
- Synonyms:
- 1.Albocarbon
- 2.Dezodorator
- 3.Moth flakes
- 4.Naphthalin
- 5.Naphthaline
- 6.Naphthene
- 7.Tar camphor
- 8.White tar
- 9.Camphor tar
- 10.Moth balls
- 11.Naftalen
- 12.NCI-C52904
- 13.Mighty 150
- 14.Mighty RD1
- 15.Rcra waste number U165
- 16.UN 1334
- 17.UN 2304
- 18.NSC 37565

- Estimated non-polar retention index (n-alkane scale):
- Value: 1231 iu
- Confidence interval (Aromatic Hydrocarbons): 55(50%) 238(95%) iu

13.565

- Name: 1-Tetradecene
- Formula: $C_{14}H_{28}$
- MW: 196 Exact Mass: 196.219101 CAS#: 1120-36-1 NIST#: 69725 ID#: 1735 DB: replib
- Other DBs: Fine, TSCA, HODOC, NIH, EINECS, IRDB
- Contributor: MASS SPECTRA OF ORGANIC COMPOUNDS, CSIRO, B.H. KENNETT ET AL
- 10 largest peaks:
- 43 999 | 41 920 | 55 810 | 57 650 | 56 570 |
- 69 550 | 83 530 | 70 500 | 29 490 | 97 360 |
- Synonyms:
- 1.n-Tetradec-1-ene
- 2.α-Tetradecene
- 3.Neodene 14
- 4.Tetradec-1-ene
- 5.Tetradecene-1

- Estimated non-polar retention index (n-alkane scale):
- Value: 1403 iu
- Confidence interval (Hydrocarbons): 39(50%) 167(95%) iu

16.032

- Name: Cetene
- Formula: $C_{16}H_{32}$
- MW: 224 Exact Mass: 224.2504015 CAS#: 629-73-2 NIST#: 69727 ID#: 5717 DB: mainlib
- Other DBs: Fine, TSCA, RTECS, EPA, HODOC, NIH, EINECS, IRDB
- Contributor: MASS SPECTRA OF ORGANIC COMPOUNDS, CSIRO, B.H. KENNETT ET AL
- Related CAS#: 501012-99-3
- 10 largest peaks:
- 43 999 | 41 870 | 55 850 | 57 830 | 69 590 |
- 83 590 | 56 560 | 97 460 | 29 450 | 70 450 |
- Synonyms:
- 1.1-Hexadecene
- 2.α-Hexadecene
- 3.n-Hexadec-1-ene
- 4.1-Cetene
- 5.Hexadecylene-1
- 6.Hexadec-1-ene
- 7.Hexadecene-1
- 8.Neodene 16
- 9.1-n-Hexadecene
- 10.NSC 60602

- Estimated non-polar retention index (n-alkane scale):
- Value: 1602 iu
- Confidence interval (Hydrocarbons): 39(50%) 167(95%) iu

Sample SM

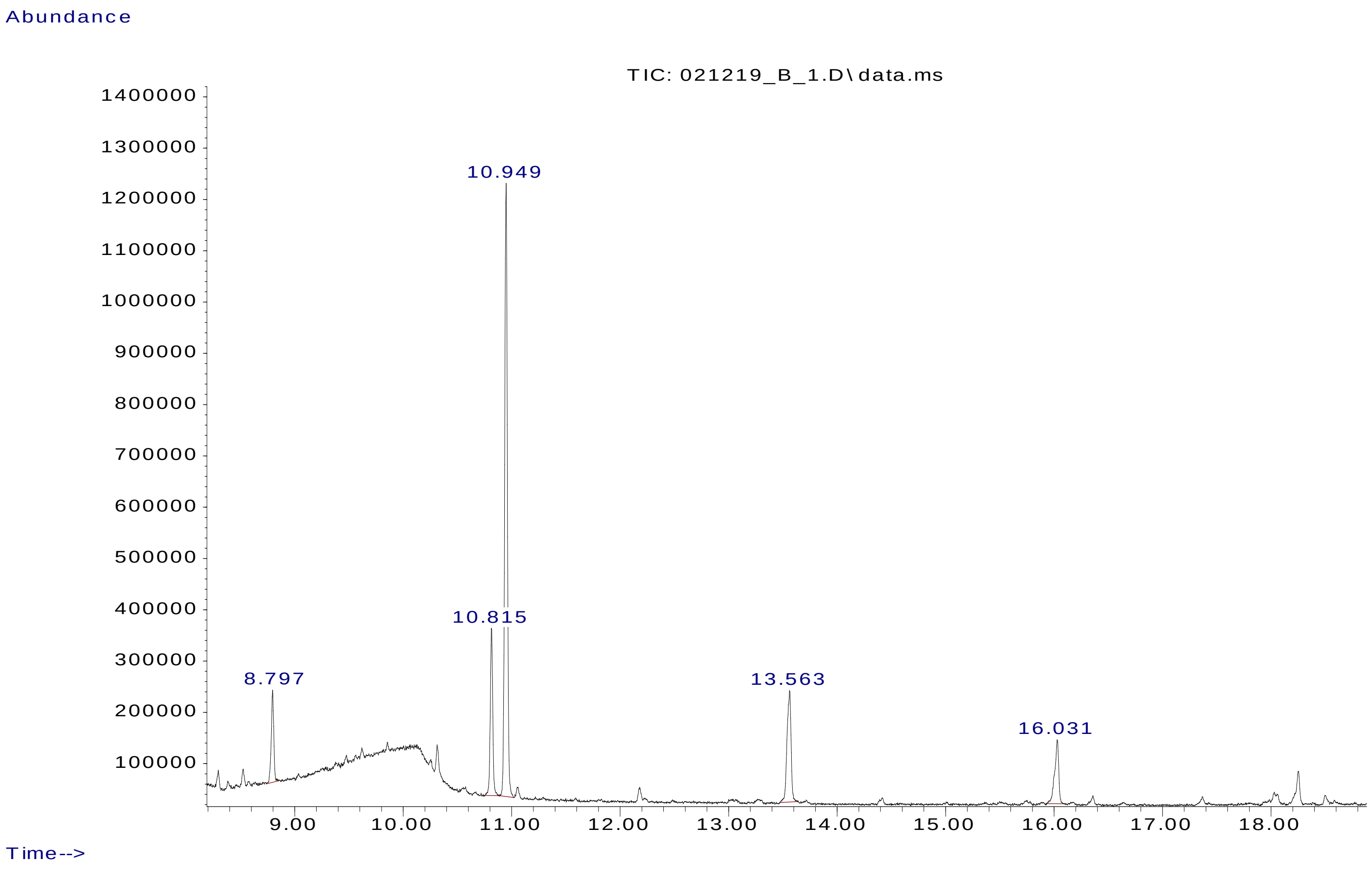
Abundance
TIC: 021219_B_1.D\data.ms
1400000
1300000
1200000
1100000
1000000
900000
800000
700000
600000
500000
400000
300000
200000
100000
10.949
10.815
8.797
13.563
16.031
9.00
10.00
11.00
12.00
13.00
14.00
15.00
16.00
17.00
18.00
Time-->

- Data Path : G:\GC-MS data\IISc\2019\Rashmi\
- Data File : 021219_B_1.D
- Acq On : 29 Feb 2020 20:27 (#1); 29 Feb 2020 20:19 (#2)
- Operator : Muralidhar
- Sample : 021219_B_1
- Misc :
- ALS Vial : 2 Sample Multiplier: 1

- Integration Parameters: autoint1.e
- Integrator: ChemStation

- Method : D:\GCMS\IISc\2016\BC\CJB\Hex July yogi\130716_SA1.D\2016_KAMALRAJ_SPLIT25.M
- Title :

- Signal : TIC: 021219_B_1.D\data.ms

peak #	R.T. min	first scan	max scan	last scan	PK TY	peak height	corr. area	corr. % max.	% of total
1	8.797	163	179	195	BB 2	177790	2919599	14.13%	7.749%
2	10.815	752	773	792	BB 3	326602	5234276	25.32%	13.893%
3	10.949	794	812	833	BV	1189807	20669068	100.00%	54.862%
4	13.563	1554	1581	1601	BB 3	216585	5607522	27.13%	14.884%
5	16.031	2277	2306	2323	BB 4	124506	3244460	15.70%	8.612%

- Sum of corrected areas: 37674923
- Signal : 021219_B_1.D\FID1A.ch

peak #	R.T. min	Start min	End min	PK TY	peak height	corr. area	corr. % max.	% of total
1	3.003	2.281	3.075	BV	52390416	16996987311	63.11%	30.107%
2	3.323	3.075	3.395	VV	52729656	9795731343	36.37%	17.352%
3	4.178	3.395	4.288	VB	61189469	26932157400	100.00%	47.706%
4	5.431	4.881	5.635	BB	15213184	2729760841	10.14%	4.835%

- Sum of corrected areas: 56454636895

- 2016_KAMALRAJ_SPLIT25.M Fri Dec 06 15:59:18 2019

Peak1 8.797

- Formula: $C_9H_{22}O_3Si_2$
- MW: 234 Exact Mass: 234.110747 CAS#: 17596-96-2 NIST#: 78865 ID#: 38429 DB: mainlib
- Other DBs: None
- Contributor: O A MAMER, MCGILL UNIVERSITY, MONTREAL, CANADA
- 10 largest peaks:
- 73 999 | 117 696 | 147 691 | 45 182 | 75 142 |
- 191 126 | 66 122 | 148 112 | 190 99 | 74 92 |
- Synonyms:
- 1.Propionic acid, 2-(trimethylsiloxy)-, trimethylsilyl ester
- 2.Bis(trimethylsilyl)lactate
- 3.Lactic acid, bis(trimethylsilyl)oxy-, ester
- 4.2-Trimethylsilyloxypropionic acid,trimethylsilyl ester
- 5.Trimethylsilyl 2-[(trimethylsilyl)oxy]propanoate
- 6.Lactic acid, bis-TMS
- 7.Lactic acid, di-TMS
- 8.Lactic acid, O,O-TMS
- 9.Lactic acid, (2TMS)

- Estimated non-polar retention index (n-alkane scale):
- Value: 915 iu
- Confidence interval (Diverse functional groups): 89(50%) 382(95%) iu

Peak 2 10.815

- Name: Cyclopropane, nonyl-
- Formula: $C_{12}H_{24}$
- MW: 168 Exact Mass: 168.1878 CAS#: 74663-85-7 NIST#: 62608 ID#: 18566 DB: mainlib
- Other DBs: None
- Contributor: D.HENNEBERG, MAX-PLANCK INSTITUTE, MULHEIM, WEST GERMANY
- 10 largest peaks:
- 55 999 | 56 912 | 43 850 | 69 733 | 41 709 |
- 70 673 | 57 568 | 83 507 | 29 402 | 97 315 |
- Synonyms:
- 1.Nonylcyclopropane #
- 2.n-Nonyl-cyclopropane

- Estimated non-polar retention index (n-alkane scale):
- Value: 1216 iu
- Confidence interval (Hydrocarbons): 39(50%) 167(95%) iu

- Retention index.
- 1. Value: 1285 iu
- Column Type: Capillary
- Column Class: Semi-standard non-polar
- Active Phase: DB-5
- Column Length: 30 m
- Carrier Gas: He
- Column Diameter: 0.13 mm
- Data Type: Kovats RI
- Program Type: Ramp
- Start T: 30 C
- End T: 280 C
- Heat Rate: 10 K/min
- Start Time: 2 min
- End Time: 3 min
- Source: Ramarathnam, N.; Rubin, L.J.; Diosady, L.L., **Studies on meat flavor. 3. A novel method for trapping volatile components from uncured and cured pork,** ***J. Agric. Food Chem., 41(6), 1993, 933-938.***

- 2. Value: 1285 iu
- Column Type: Capillary
- Column Class: Semi-standard non-polar
- Active Phase: DB-5
- Column Length: 30 m
- Carrier Gas: He
- Column Diameter: 0.13 mm
- Data Type: Kovats RI
- Program Type: Ramp
- Start T: 30 C
- End T: 280 C
- Heat Rate: 10 K/min
- Start Time: 2 min
- End Time: 3 min
- Source: Ramarathnam, N.; Rubin, L.J.; Diosady, L.L., **Studies on meat flavor. 4. Fractionation, characterization, and quantitation of volatiles from uncured and cured beef and chicken,** ***J. Agric. Food Chem., 41(6), 1993, 939-945.***

- <...>

Peak 3 10.949

- Name: Naphthalene
- Formula: $C_{10}H_8$
- MW: 128 Exact Mass: 128.0626 CAS#: 91-20-3 NIST#: 379701 ID#: 19026 DB: replib
- Other DBs: Fine, TSCA, RTECS, EPA, HODOC, NIH, EINECS, IRDB
- Contributor: Drug Lab
- Related CAS#: 72931-45-4
- 10 largest peaks:
- 128 999 | 127 126 | 129 108 | 102 85 | 126 72 |
- 51 54 | 63 45 | 64 45 | 75 36 | 74 34 |
- Synonyms:
- 1.Albocarbon
- 2.Dezodorator
- 3.Moth flakes
- 4.Naphthalin
- 5.Naphthaline
- 6.Naphthene
- 7.Tar camphor
- 8.White tar
- 9.Camphor tar
- 10.Moth balls
- 11.Naftalen
- 12.NCI-C52904
- 13.Mighty 150
- 14.Mighty RD1
- 15.Rcra waste number U165
- 16.UN 1334
- 17.UN 2304
- 18.NSC 37565
- Estimated non-polar retention index (n-alkane scale):
- Value: 1231 iu
- Confidence interval (Aromatic Hydrocarbons): 55(50%) 238(95%) iu

Peak 4 13.563

- Name: Cyclotetradecane
- Formula: $C_{14}H_{28}$
- MW: 196 Exact Mass: 196.219101 CAS#: 295-17-0 NIST#: 61052 ID#: 4454 DB: replib
- Other DBs: EINECS, IRDB
- Contributor: D.HENNEBERG, MAX-PLANCK INSTITUTE, MULHEIM, WEST GERMANY
- 10 largest peaks:
- 55 999 | 41 882 | 69 679 | 43 641 | 83 612 |
- 56 597 | 57 529 | 70 436 | 97 393 | 29 356 |
- Synonyms:
- no synonyms.

- Estimated non-polar retention index (n-alkane scale):
- Value: 1679 iu
- Confidence interval (Hydrocarbons): 39(50%) 167(95%) iu

Peak 5 13.061

- Name: Cetene
- Formula: $C_{16}H_{32}$
- MW: 224 Exact Mass: 224.2504015 CAS#: 629-73-2 NIST#: 69727 ID#: 5717 DB: mainlib
- Other DBs: Fine, TSCA, RTECS, EPA, HODOC, NIH, EINECS, IRDB
- Contributor: MASS SPECTRA OF ORGANIC COMPOUNDS, CSIRO, B.H. KENNETT ET AL
- Related CAS#: 501012-99-3
- 10 largest peaks:
- 43 999 | 41 870 | 55 850 | 57 830 | 69 590 |
- 83 590 | 56 560 | 97 460 | 29 450 | 70 450 |
- Synonyms:
- 1.1-Hexadecene
- 2.α-Hexadecene
- 3.n-Hexadec-1-ene
- 4.1-Cetene
- 5.Hexadecylene-1
- 6.Hexadec-1-ene
- 7.Hexadecene-1
- 8.Neodene 16
- 9.1-n-Hexadecene
- 10.NSC 60602

- Estimated non-polar retention index (n-alkane scale):
- Value: 1602 iu
- Confidence interval (Hydrocarbons): 39(50%) 167(95%) iu

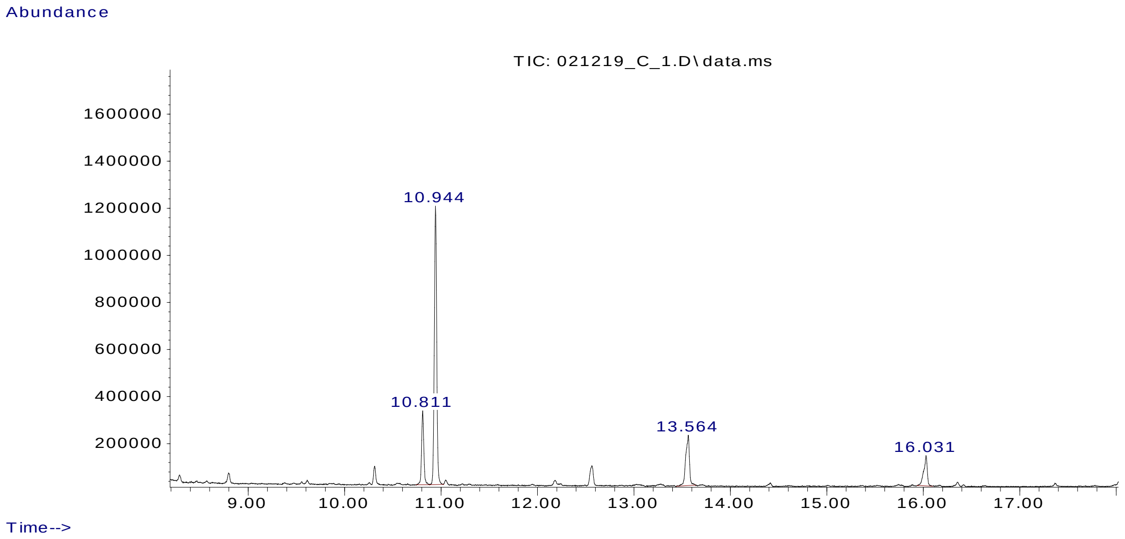
Abundance
TIC: 021219_C_1.D\data.ms
1600000
1400000
1200000
1000000
800000
600000
400000
200000
10.944
10.811
13.564
16.031
9.00
10.00
11.00
12.00
13.00
14.00
15.00
16.00
17.00
Time-->

- GCMS facility_DBS Area Percent Report

- Data Path : G:\GC-MS data\IISc\2019\Rashmi\
- Data File : 021219_C_1.D
- Acq On : 29 Feb 2020 21:00 (#1); 29 Feb 2020 20:52 (#2)
- Operator : Muralidhar
- Sample : 021219_C_1
- Misc :
- ALS Vial : 3 Sample Multiplier: 1

- Integration Parameters: autoint1.e
- Integrator: ChemStation

- Method : D:\GCMS\IISc\2016\BC\CJB\Hex July yogi\130716_SA1.D\2016_KAMALRAJ_SPLIT25.M
- Title :

- Signal : TIC: 021219_C_1.D\data.ms

```
peak  R.T. first  max last  PK   peak      corr.   corr.    % of
  #   min   scan scan scan  TY  height     area    % max.   total
---  ----- ----- ---- ---- ---  -------   -------  ------  -------
  1  10.811   751  771  792 BB 2  310349   5158751  25.02%  14.867%
  2  10.944   794  811  834 BV   1174621  20616664 100.00%  59.414%
  3  13.564  1545 1581 1603 BB 3  214181   5696205  27.63%  16.416%
  4  16.031  2278 2306 2324 BB 3  128834   3228156  15.66%   9.303%
```

- Sum of corrected areas: 34699776
- Signal : 021219_C_1.D\FID1A.ch

```
peak  R.T.   Start    End   PK  peak       corr.    corr.    % of
  #   min     min     min   TY height      area     % max.   total
---  -----   -----   -----  --- -------   -------  ------  ------
  1  3.004   2.272   3.088  BV  54104517 17611932803  48.27% 31.432%
  2  4.180   3.088   4.285  VB  61063858 36486051105 100.00% 65.116%
  3  5.480   5.092   5.655  BB  13877032 1934507322   5.30%   3.452%
```

- Sum of corrected areas: 56032491229

- 2016_KAMALRAJ_SPLIT25.M Fri Dec 06 16:04:40 2019

Peak 6 10.811

- Name: 1-Dodecene
- Formula: $C_{12}H_{24}$
- MW: 168 Exact Mass: 168.1878 CAS#: 112-41-4 NIST#: 107688 ID#: 4383 DB: replib
- Other DBs: Fine, TSCA, HODOC, NIH, EINECS, IRDB
- Contributor: Chuck Anderson, Aldrich Chemical Co.
- 10 largest peaks:
- 55 999 | 41 931 | 43 905 | 56 803 | 83 666 |
- 69 572 | 70 536 | 97 527 | 57 523 | 84 414 |
- Synonyms:
- 1.α-Dodecene
- 2.n-Dodec-1-ene
- 3.Adacene 12
- 4.Dodec-1-ene
- 5.α-Dodecylene
- 6.Dodecylene α-
- 7.Dodecene-1
- 8.Neodene 12
- 9.NSC 12016

- Estimated non-polar retention index (n-alkane scale):
- Value: 1204 iu
- Confidence interval (Hydrocarbons): 39(50%) 167(95%) iu

Peak 7 10.944

- Name: Naphthalene
- Formula: $C_{10}H_8$
- MW: 128 Exact Mass: 128.0626 CAS#: 91-20-3 NIST#: 379701 ID#: 19026 DB: replib
- Other DBs: Fine, TSCA, RTECS, EPA, HODOC, NIH, EINECS, IRDB
- Contributor: Drug Lab
- Related CAS#: 72931-45-4
- 10 largest peaks:
- 128 999 | 127 126 | 129 108 | 102 85 | 126 72 |
- 51 54 | 63 45 | 64 45 | 75 36 | 74 34 |
- Synonyms:
- 1.Albocarbon
- 2.Dezodorator
- 3.Moth flakes
- 4.Naphthalin
- 5.Naphthaline
- 6.Naphthene
- 7.Tar camphor
- 8.White tar
- 9.Camphor tar
- 10.Moth balls
- 11.Naftalen
- 12.NCI-C52904
- 13.Mighty 150
- 14.Mighty RD1
- 15.Rcra waste number U165
- 16.UN 1334
- 17.UN 2304
- 18.NSC 37565
- Estimated non-polar retention index (n-alkane scale):
- Value: 1231 iu
- Confidence interval (Aromatic Hydrocarbons): 55(50%) 238(95%) iu

Peak 8 13.564

- Name: 1-Tetradecene
- Formula: $C_{14}H_{28}$
- MW: 196 Exact Mass: 196.219101 CAS#: 1120-36-1 NIST#: 69725 ID#: 1735 DB: replib
- Other DBs: Fine, TSCA, HODOC, NIH, EINECS, IRDB
- Contributor: MASS SPECTRA OF ORGANIC COMPOUNDS, CSIRO, B.H. KENNETT ET AL
- 10 largest peaks:
- 43 999 | 41 920 | 55 810 | 57 650 | 56 570 |
- 69 550 | 83 530 | 70 500 | 29 490 | 97 360 |
- Synonyms:
- 1.n-Tetradec-1-ene
- 2.α-Tetradecene
- 3.Neodene 14
- 4.Tetradec-1-ene
- 5.Tetradecene-1

- Estimated non-polar retention index (n-alkane scale):
- Value: 1403 iu
- Confidence interval (Hydrocarbons): 39(50%) 167(95%) iu

Peak 9 16.031

- Name: Cetene
- Formula: $C_{16}H_{32}$
- MW: 224 Exact Mass: 224.2504015 CAS#: 629-73-2 NIST#: 69727 ID#: 5717 DB: mainlib
- Other DBs: Fine, TSCA, RTECS, EPA, HODOC, NIH, EINECS, IRDB
- Contributor: MASS SPECTRA OF ORGANIC COMPOUNDS, CSIRO, B.H. KENNETT ET AL
- Related CAS#: 501012-99-3
- 10 largest peaks:
- 43 999 | 41 870 | 55 850 | 57 830 | 69 590 |
- 83 590 | 56 560 | 97 460 | 29 450 | 70 450 |
- Synonyms:
- 1.1-Hexadecene
- 2.α-Hexadecene
- 3.n-Hexadec-1-ene
- 4.1-Cetene
- 5.Hexadecylene-1
- 6.Hexadec-1-ene
- 7.Hexadecene-1
- 8.Neodene 16
- 9.1-n-Hexadecene
- 10.NSC 60602

- Estimated non-polar retention index (n-alkane scale):
- Value: 1602 iu
- Confidence interval (Hydrocarbons): 39(50%) 167(95%) iu

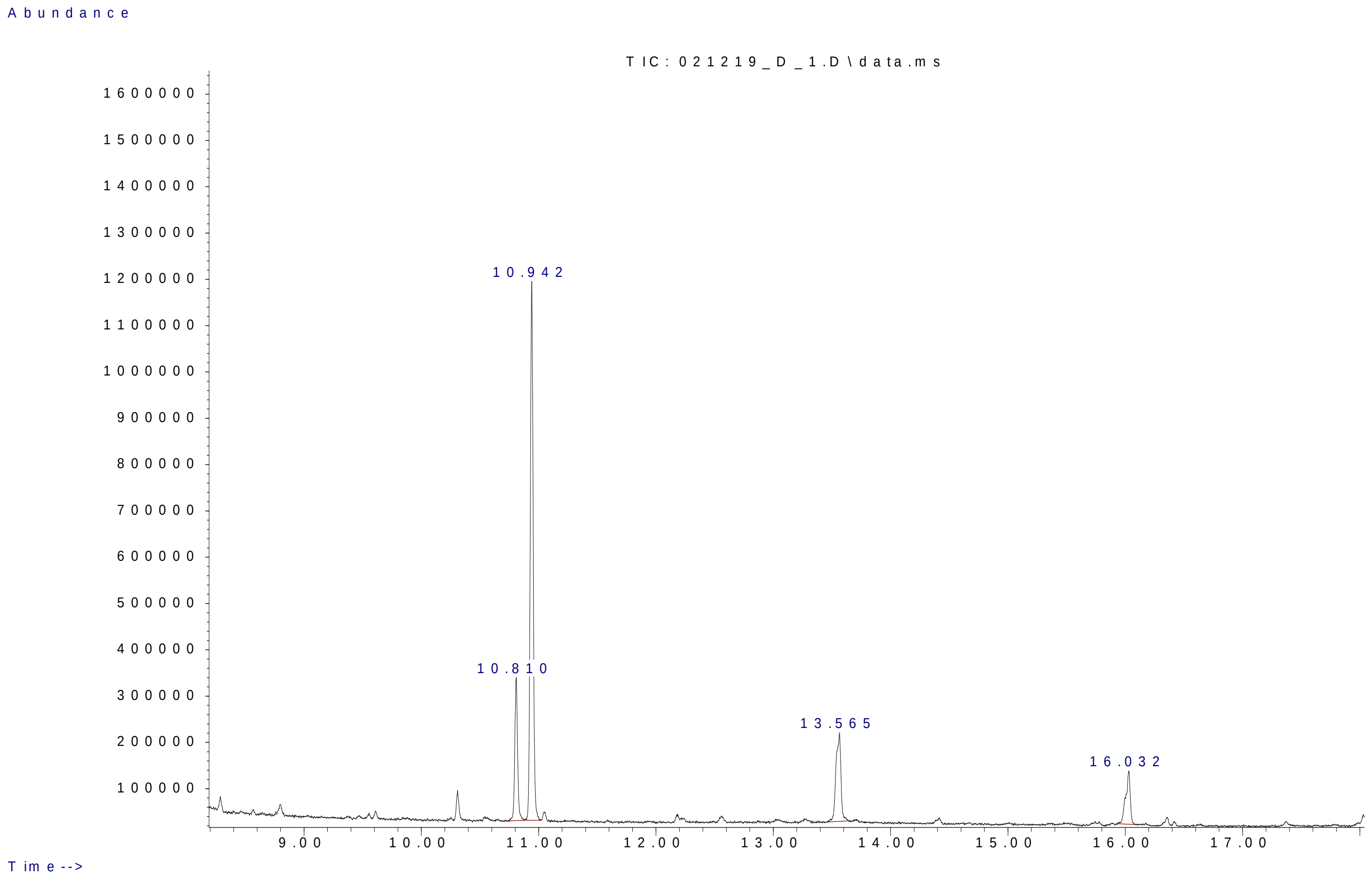
Abundance
TIC: 021219_D_1.D\data.ms
1600000
1500000
1400000
1300000
1200000
1100000
1000000
900000
800000
700000
600000
500000
400000
300000
200000
100000
10.942
10.810
13.565
16.032
9.00
10.00
11.00
12.00
13.00
14.00
15.00
16.00
17.00
Time-->

- Data Path : G:\GC-MS data\IISc\2019\Rashmi\
- Data File : 021219_D_1.D
- Acq On : 29 Feb 2020 21:34 (#1); 29 Feb 2020 21:26 (#2)
- Operator : Muralidhar
- Sample : 021219_D_1
- Misc :
- ALS Vial : 4 Sample Multiplier: 1

- Integration Parameters: autoint1.e
- Integrator: ChemStation

- Method : D:\GCMS\IISc\2016\BC\CJB\Hex July yogi\130716_SA1.D\2016_KAMALRAJ_SPLIT25.M
- Title :

- Signal : TIC: 021219_D_1.D\data.ms

- peak R.T. first max last PK peak corr. corr. % of
- # min scan scan scan TY height area % max. total
- --- ----- ----- ---- ---- --- ------- ------- ------ -------
- 1 10.810 747 771 793 BB 2 307477 5131162 25.12% 14.994%
- 2 10.942 796 810 831 BV 1156860 20424735 100.00% 59.682%
- 3 13.565 1549 1581 1604 BB 4 190005 5590327 27.37% 16.335%
- 4 16.032 2277 2306 2324 BB 3 115010 3076196 15.06% 8.989%
-
-
- Sum of corrected areas: 34222419
- Signal : 021219_D_1.D\FID1A.ch

- peak R.T. Start End PK peak corr. corr. % of
- # min min min TY height area % max. total
- --- ----- ----- ----- --- ------- ------- ------ ------
- 1 3.010 2.281 3.083 BV 56176470 17611505240 74.05% 31.495%
- 2 3.764 3.083 3.828 VV 55872130 23783509614 100.00% 42.533%
- 3 4.169 3.828 4.265 VB 60927744 12475007724 52.45% 22.310%
- 4 5.454 4.831 5.500 BV 11715227 2047781197 8.61% 3.662%
-
-
- Sum of corrected areas: 55917803775

- 2016_KAMALRAJ_SPLIT25.M Fri Dec 06 16:10:33 2019

Peak 10 10.810

- Name: 1-Dodecene
- Formula: $C_{12}H_{24}$
- MW: 168 Exact Mass: 168.1878 CAS#: 112-41-4 NIST#: 107688 ID#: 4383 DB: replib
- Other DBs: Fine, TSCA, HODOC, NIH, EINECS, IRDB
- Contributor: Chuck Anderson, Aldrich Chemical Co.
- 10 largest peaks:
- 55 999 | 41 931 | 43 905 | 56 803 | 83 666 |
- 69 572 | 70 536 | 97 527 | 57 523 | 84 414 |
- Synonyms:
- 1.α-Dodecene
- 2.n-Dodec-1-ene
- 3.Adacene 12
- 4.Dodec-1-ene
- 5.α-Dodecylene
- 6.Dodecylene α-
- 7.Dodecene-1
- 8.Neodene 12
- 9.NSC 12016

- Estimated non-polar retention index (n-alkane scale):
- Value: 1204 iu
- Confidence interval (Hydrocarbons): 39(50%) 167(95%) iu

Peak 11 10.942

- Name: Naphthalene
- Formula: $C_{10}H_8$
- MW: 128 Exact Mass: 128.0626 CAS#: 91-20-3 NIST#: 379701 ID#: 19026 DB: replib
- Other DBs: Fine, TSCA, RTECS, EPA, HODOC, NIH, EINECS, IRDB
- Contributor: Drug Lab
- Related CAS#: 72931-45-4
- 10 largest peaks:
- 128 999 | 127 126 | 129 108 | 102 85 | 126 72 |
- 51 54 | 63 45 | 64 45 | 75 36 | 74 34 |
- Synonyms:
- 1.Albocarbon
- 2.Dezodorator
- 3.Moth flakes
- 4.Naphthalin
- 5.Naphthaline
- 6.Naphthene
- 7.Tar camphor
- 8.White tar
- 9.Camphor tar
- 10.Moth balls
- 11.Naftalen
- 12.NCI-C52904
- 13.Mighty 150
- 14.Mighty RD1
- 15.Rcra waste number U165
- 16.UN 1334
- 17.UN 2304
- 18.NSC 37565
- Estimated non-polar retention index (n-alkane scale):
- Value: 1231 iu
- Confidence interval (Aromatic Hydrocarbons): 55(50%) 238(95%) iu

Peak 12 13.565

- Name: 1-Tetradecene
- Formula: $C_{14}H_{28}$
- MW: 196 Exact Mass: 196.219101 CAS#: 1120-36-1 NIST#: 69725 ID#: 1735 DB: replib
- Other DBs: Fine, TSCA, HODOC, NIH, EINECS, IRDB
- Contributor: MASS SPECTRA OF ORGANIC COMPOUNDS, CSIRO, B.H. KENNETT ET AL
- 10 largest peaks:
- 43 999 | 41 920 | 55 810 | 57 650 | 56 570 |
- 69 550 | 83 530 | 70 500 | 29 490 | 97 360 |
- Synonyms:
- 1.n-Tetradec-1-ene
- 2.α-Tetradecene
- 3.Neodene 14
- 4.Tetradec-1-ene
- 5.Tetradecene-1

- Estimated non-polar retention index (n-alkane scale):
- Value: 1403 iu
- Confidence interval (Hydrocarbons): 39(50%) 167(95%) iu

Peak 13 16.032

- Name: Cetene
- Formula: $C_{16}H_{32}$
- MW: 224 Exact Mass: 224.2504015 CAS#: 629-73-2 NIST#: 69727 ID#: 5717 DB: mainlib
- Other DBs: Fine, TSCA, RTECS, EPA, HODOC, NIH, EINECS, IRDB
- Contributor: MASS SPECTRA OF ORGANIC COMPOUNDS, CSIRO, B.H. KENNETT ET AL
- Related CAS#: 501012-99-3
- 10 largest peaks:
- 43 999 | 41 870 | 55 850 | 57 830 | 69 590 |
- 83 590 | 56 560 | 97 460 | 29 450 | 70 450 |
- Synonyms:
- 1.1-Hexadecene
- 2.α-Hexadecene
- 3.n-Hexadec-1-ene
- 4.1-Cetene
- 5.Hexadecylene-1
- 6.Hexadec-1-ene
- 7.Hexadecene-1
- 8.Neodene 16
- 9.1-n-Hexadecene
- 10.NSC 60602

- Estimated non-polar retention index (n-alkane scale):
- Value: 1602 iu
- Confidence interval (Hydrocarbons): 39(50%) 167(95%) iu

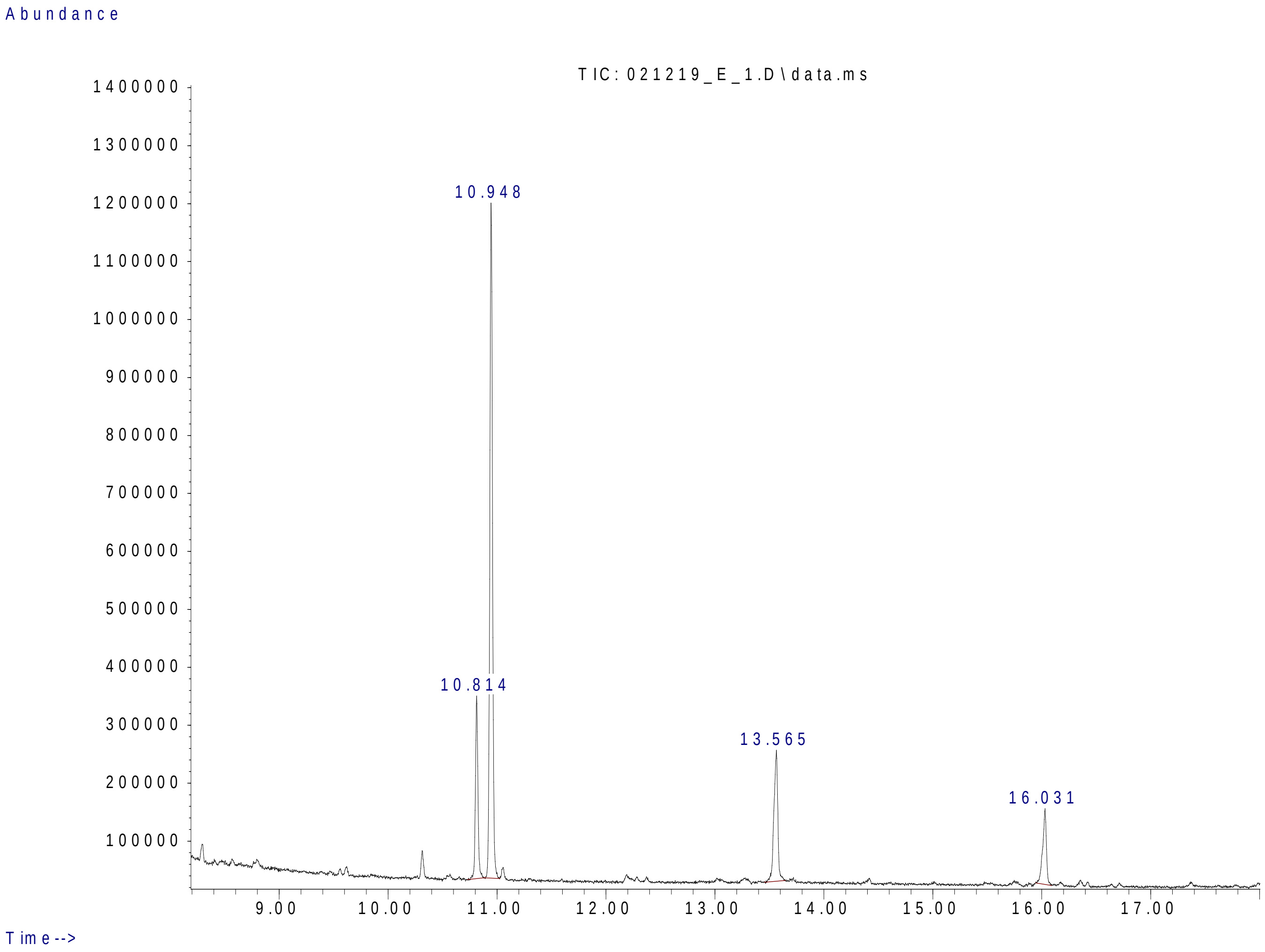

Abundance
TIC: 021219_E_1.D\data.ms
1400000
1300000
1200000
1100000
1000000
900000
800000
700000
600000
500000
400000
300000
200000
100000
10.948
10.814
13.565
16.031
9.00
10.00
11.00
12.00
13.00
14.00
15.00
16.00
17.00
Time-->

- Data Path : G:\GC-MS data\IISc\2019\Rashmi\
- Data File : 021219_E_1.D
- Acq On : 29 Feb 2020 22:08 (#1); 29 Feb 2020 22:00 (#2)
- Operator : Muralidhar
- Sample : 021219_E_1
- Misc :
- ALS Vial : 5 Sample Multiplier: 1

- Integration Parameters: autoint1.e
- Integrator: ChemStation

- Method : D:\GCMS\IISc\2016\BC\CJB\Hex July yogi\130716_SA1.D\2016_KAMALRAJ_SPLIT25.M
- Title :

- Signal : TIC: 021219_E_1.D\data.ms

peak #	R.T. min	first scan	max scan	last scan	PK TY	peak height	corr. area	corr. % max.	% of total
1	10.814	747	772	791	BB 2	312913	5041697	24.69%	14.759%
2	10.948	794	812	835	BV	1157589	20418260	100.00%	59.772%
3	13.565	1550	1581	1604	BB 3	223986	5609718	27.47%	16.422%
4	16.031	2281	2306	2323	BB 4	129270	3090457	15.14%	9.047%

- Sum of corrected areas: 34160131
- Signal : 021219_E_1.D\FID1A.ch

peak #	R.T. min	Start min	End min	PK TY	peak height	corr. area	corr. % max.	% of total
1	3.016	2.288	3.083	BV	14527814	6599122837	51.40%	30.775%
2	3.118	3.083	3.148	VB	2451091	86154014	0.67%	0.402%
3	4.170	3.415	4.261	BB	55548267	12839459986	100.00%	59.876%
4	5.286	5.021	5.405	BV	10594362	1306464339	10.18%	6.093%
5	5.457	5.405	5.521	VV	11409617	612133475	4.77%	2.855%

- Sum of corrected areas: 21443334652

- 2016_KAMALRAJ_SPLIT25.M Fri Dec 06 16:14:21 2019

Peak 14 10.814

- Name: Cyclopropane, nonyl-
- Formula: $C_{12}H_{24}$
- MW: 168 Exact Mass: 168.1878 CAS#: 74663-85-7 NIST#: 62608 ID#: 18566 DB: mainlib
- Other DBs: None
- Contributor: D.HENNEBERG, MAX-PLANCK INSTITUTE, MULHEIM, WEST GERMANY
- 10 largest peaks:
- 55 999 | 56 912 | 43 850 | 69 733 | 41 709 |
- 70 673 | 57 568 | 83 507 | 29 402 | 97 315 |
- Synonyms:
- 1.Nonylcyclopropane #
- 2.n-Nonyl-cyclopropane

- Estimated non-polar retention index (n-alkane scale):
- Value: 1216 iu
- Confidence interval (Hydrocarbons): 39(50%) 167(95%) iu

Peak 15 10.948

- Name: Naphthalene
- Formula: $C_{10}H_8$
- MW: 128 Exact Mass: 128.0626 CAS#: 91-20-3 NIST#: 379701 ID#: 19026 DB: replib
- Other DBs: Fine, TSCA, RTECS, EPA, HODOC, NIH, EINECS, IRDB
- Contributor: Drug Lab
- Related CAS#: 72931-45-4
- 10 largest peaks:
- 128 999 | 127 126 | 129 108 | 102 85 | 126 72 |
- 51 54 | 63 45 | 64 45 | 75 36 | 74 34 |
- Synonyms:
- 1.Albocarbon
- 2.Dezodorator
- 3.Moth flakes
- 4.Naphthalin
- 5.Naphthaline
- 6.Naphthene
- 7.Tar camphor
- 8.White tar
- 9.Camphor tar
- 10.Moth balls
- 11.Naftalen
- 12.NCI-C52904
- 13.Mighty 150
- 14.Mighty RD1
- 15.Rcra waste number U165
- 16.UN 1334
- 17.UN 2304
- 18.NSC 37565
- Estimated non-polar retention index (n-alkane scale):
- Value: 1231 iu
- Confidence interval (Aromatic Hydrocarbons): 55(50%) 238(95%) iu

Peak 16 13.565

- Name: 1-Tetradecene
- Formula: $C_{14}H_{28}$
- MW: 196 Exact Mass: 196.219101 CAS#: 1120-36-1 NIST#: 69725 ID#: 1735 DB: replib
- Other DBs: Fine, TSCA, HODOC, NIH, EINECS, IRDB
- Contributor: MASS SPECTRA OF ORGANIC COMPOUNDS, CSIRO, B.H. KENNETT ET AL
- 10 largest peaks:
- 43 999 | 41 920 | 55 810 | 57 650 | 56 570 |
- 69 550 | 83 530 | 70 500 | 29 490 | 97 360 |
- Synonyms:
- 1.n-Tetradec-1-ene
- 2.α-Tetradecene
- 3.Neodene 14
- 4.Tetradec-1-ene
- 5.Tetradecene-1

- Estimated non-polar retention index (n-alkane scale):
- Value: 1403 iu
- Confidence interval (Hydrocarbons): 39(50%) 167(95%) iu

Peak 17 16.031

- Name: 1-Tetradecene
- Formula: $C_{14}H_{28}$
- MW: 196 Exact Mass: 196.219101 CAS#: 1120-36-1 NIST#: 69725 ID#: 1735 DB: replib
- Other DBs: Fine, TSCA, HODOC, NIH, EINECS, IRDB
- Contributor: MASS SPECTRA OF ORGANIC COMPOUNDS, CSIRO, B.H. KENNETT ET AL
- 10 largest peaks:
- 43 999 | 41 920 | 55 810 | 57 650 | 56 570 |
- 69 550 | 83 530 | 70 500 | 29 490 | 97 360 |
- Synonyms:
- 1.n-Tetradec-1-ene
- 2.α-Tetradecene
- 3.Neodene 14
- 4.Tetradec-1-ene
- 5.Tetradecene-1

- Estimated non-polar retention index (n-alkane scale):
- Value: 1403 iu
- Confidence interval (Hydrocarbons): 39(50%) 167(95%) iu

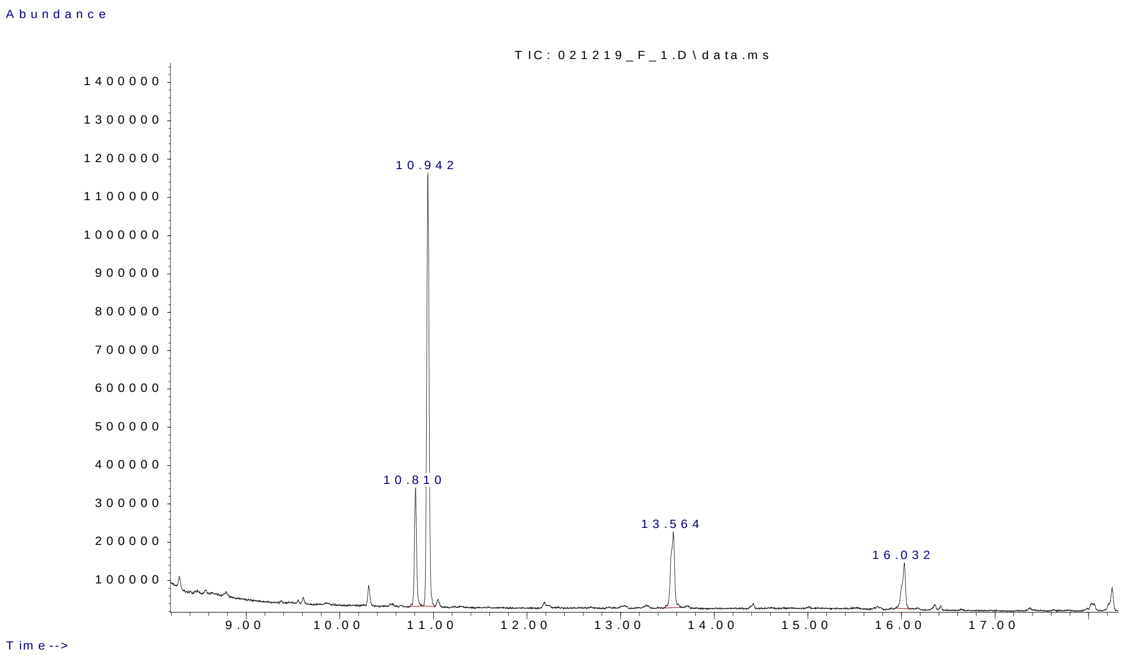
Abundance
TIC: 021219_F_1.D\data.ms
1400000
1300000
1200000
1100000
1000000
900000
800000
700000
600000
500000
400000
300000
200000
100000
10.942
10.810
13.564
16.032
9.00
10.00
11.00
12.00
13.00
14.00
15.00
16.00
17.00
Time-->

- Data Path : G:\GC-MS data\IISc\2019\Rashmi\
- Data File : 021219_F_1.D
- Acq On : 29 Feb 2020 22:42 (#1); 29 Feb 2020 22:34 (#2)
- Operator : Muralidhar
- Sample : 021219_F_1
- Misc :
- ALS Vial : 6 Sample Multiplier: 1

- Integration Parameters: autoint1.e
- Integrator: ChemStation

- Method : D:\GCMS\IISc\2016\BC\CJB\Hex July yogi\130716_SA1.D\2016_KAMALRAJ_SPLIT25.M
- Title :

- Signal : TIC: 021219_F_1.D\data.ms

- peak R.T. first max last PK peak corr. corr. % of
- # min scan scan scan TY height area % max. total
- --- ----- ----- ---- ---- --- ------- ------- ------ -------
- 1 10.810 745 771 791 BB 2 307596 5089672 24.97% 14.857%
- 2 10.942 792 810 831 BV 1129085 20384329 100.00% 59.503%
- 3 13.564 1549 1581 1605 BB 4 191736 5644827 27.69% 16.478%
- 4 16.032 2278 2306 2327 BB 5 118513 3138690 15.40% 9.162%
-
-
- Sum of corrected areas: 34257518
- Signal : 021219_F_1.D\FID1A.ch

- peak R.T. Start End PK peak corr. corr. % of
- # min min min TY height area % max. total
- --- ----- ----- ----- --- ------- ------- ------ ------
- 1 3.007 2.275 3.098 BV 22943641 8650475753 94.12% 41.729%
- 2 3.332 3.098 3.392 VB 3670672 1112912109 12.11% 5.369%
- 3 4.180 3.812 4.285 VB 54784951 9190440962 100.00% 44.334%
- 4 5.440 4.868 5.505 BV 10171921 1776316443 19.33% 8.569%
-
-
- Sum of corrected areas: 20730145266

- 2016_KAMALRAJ_SPLIT25.M Fri Dec 06 16:18:41 2019

Peak 18 10.810

- Name: 1-Dodecene
- Formula: $C_{12}H_{24}$
- MW: 168 Exact Mass: 168.1878 CAS#: 112-41-4 NIST#: 107688 ID#: 4383 DB: replib
- Other DBs: Fine, TSCA, HODOC, NIH, EINECS, IRDB
- Contributor: Chuck Anderson, Aldrich Chemical Co.
- 10 largest peaks:
- 55 999 | 41 931 | 43 905 | 56 803 | 83 666 |
- 69 572 | 70 536 | 97 527 | 57 523 | 84 414 |
- Synonyms:
- 1.α-Dodecene
- 2.n-Dodec-1-ene
- 3.Adacene 12
- 4.Dodec-1-ene
- 5.α-Dodecylene
- 6.Dodecylene α-
- 7.Dodecene-1
- 8.Neodene 12
- 9.NSC 12016

- Estimated non-polar retention index (n-alkane scale):
- Value: 1204 iu
- Confidence interval (Hydrocarbons): 39(50%) 167(95%) iu

Peak 19 10.942

- Name: Naphthalene
- Formula: $C_{10}H_8$
- MW: 128 Exact Mass: 128.0626 CAS#: 91-20-3 NIST#: 379701 ID#: 19026 DB: replib
- Other DBs: Fine, TSCA, RTECS, EPA, HODOC, NIH, EINECS, IRDB
- Contributor: Drug Lab
- Related CAS#: 72931-45-4
- 10 largest peaks:
- 128 999 | 127 126 | 129 108 | 102 85 | 126 72 |
- 51 54 | 63 45 | 64 45 | 75 36 | 74 34 |
- Synonyms:
- 1.Albocarbon
- 2.Dezodorator
- 3.Moth flakes
- 4.Naphthalin
- 5.Naphthaline
- 6.Naphthene
- 7.Tar camphor
- 8.White tar
- 9.Camphor tar
- 10.Moth balls
- 11.Naftalen
- 12.NCI-C52904
- 13.Mighty 150
- 14.Mighty RD1
- 15.Rcra waste number U165
- 16.UN 1334
- 17.UN 2304
- 18.NSC 37565
- Estimated non-polar retention index (n-alkane scale):
- Value: 1231 iu
- Confidence interval (Aromatic Hydrocarbons): 55(50%) 238(95%) iu

Peak 20 13.564

- Name: 6-Tridecene, (Z)-
- Formula: $C_{13}H_{26}$
- MW: 182 Exact Mass: 182.203451 CAS#: 6508-77-6 NIST#: 130982 ID#: 4496 DB: replib
- Other DBs: None
- Contributor: LAC, NIDDK, NIH, Bethesda, MD 20892
- 10 largest peaks:
- 55 999 | 41 785 | 69 669 | 43 631 | 56 612 |
- 70 501 | 83 443 | 57 402 | 97 295 | 84 254 |
- Synonyms:
- 1.Z-6-Tridecene
- 2.(6Z)-6-Tridecene
- 3.6-Tridecene, (Z)
- 4.cis-6-Tridecene

- Estimated non-polar retention index (n-alkane scale):
- Value: 1321 iu
- Confidence interval (Hydrocarbons): 39(50%) 167(95%) iu

Peak 21 16.032

- Name: 1-Tridecene
- Formula: $C_{13}H_{26}$
- MW: 182 Exact Mass: 182.203451 CAS#: 2437-56-1 NIST#: 107768 ID#: 4395 DB: replib
- Other DBs: Fine, TSCA, RTECS, EPA, HODOC, NIH, EINECS, IRDB
- Contributor: Chuck Anderson, Aldrich Chemical Co.
- 10 largest peaks:
- 55 999 | 41 922 | 43 873 | 83 802 | 56 711 |
- 69 654 | 57 647 | 97 605 | 70 563 | 84 411 |
- Synonyms:
- 1.n-Tridec-1-ene
- 2.1-C13H26
- 3.Tridecene-1
- 4.α-Tridecene
- 5.Tridec-1-ene

- Estimated non-polar retention index (n-alkane scale):
- Value: 1304 iu
- Confidence interval (Hydrocarbons): 39(50%) 167(95%) iu

- Retention index.

10.313

Name: Pentasiloxane, dodecamethyl-

Formula: C H O Si

MW: 384 Exa

29503 DB: re

Other DBs: T

Contributor:

10 largest pe

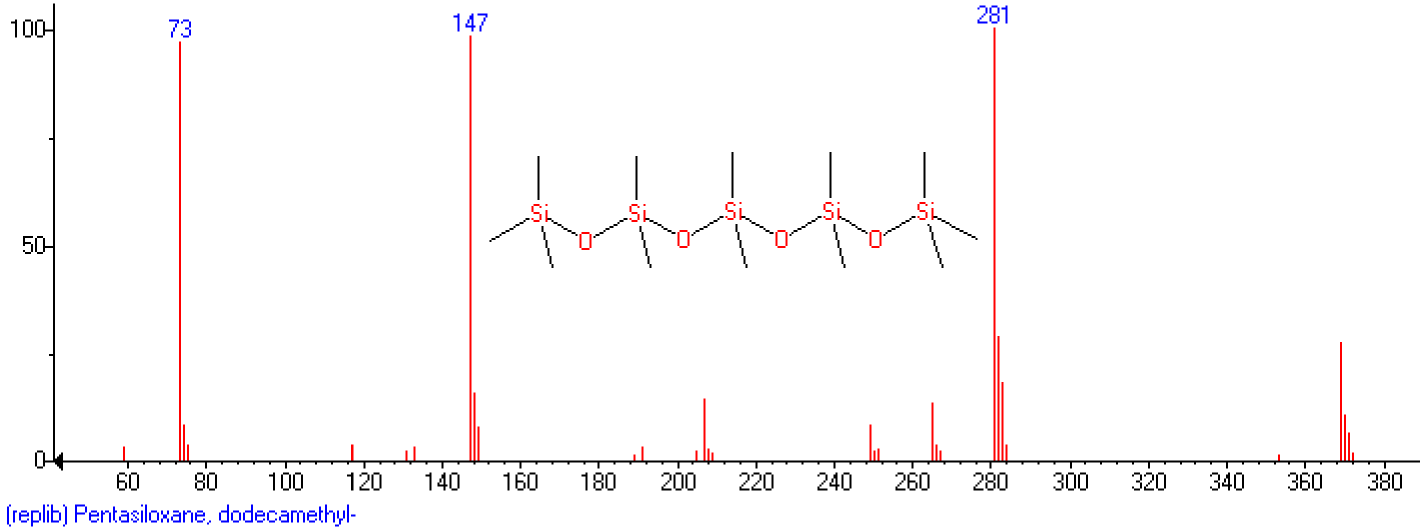

11.048

Name: Benzo[c]thiophene

Formula: C_8H_6S

MW: 134 Exact Mass: 134.019021 CAS#: 270-82-6 NIST#: 115108 ID#: 105558 DB: mainlib

Other DBs: None

Contributor: R.F.X. Klein, Dep. Chem., Georgetown Univ., Washington, D.C., USA

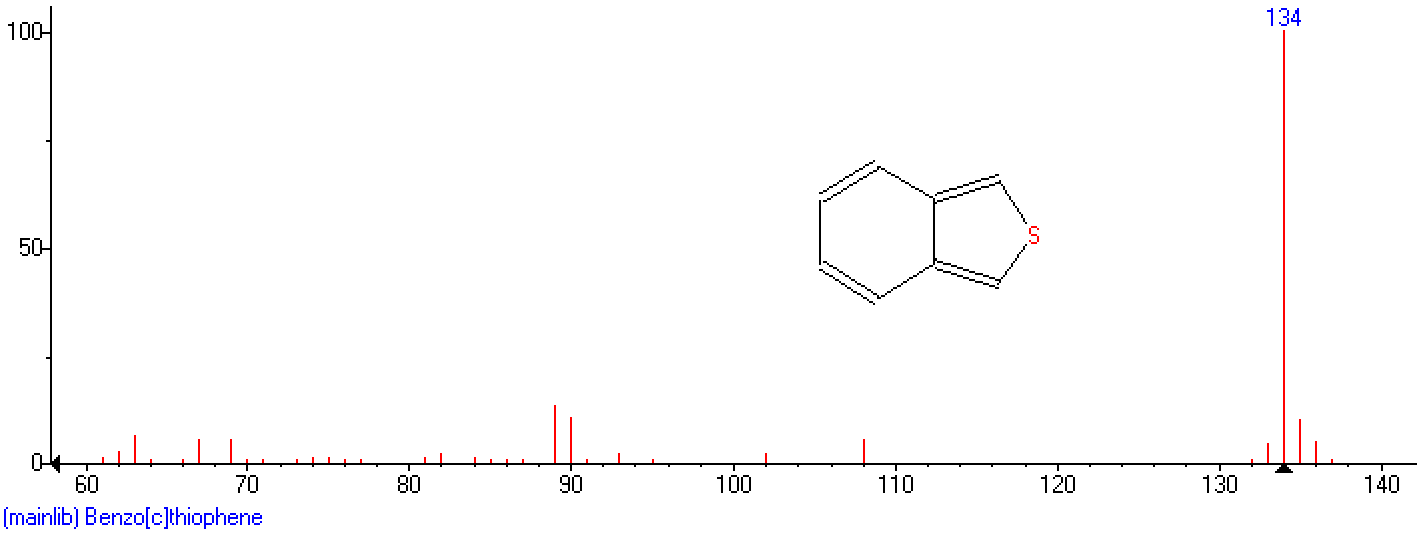

17.364

Name: 1,1'-Bicyclohexyl-1,1'-diol

Formula: $C_{12}H_{22}O_2$

MW: 198 Exact Mass: 198.16198 CAS#: 2888-11-1 NIST#: 75446 ID#: 14128 DB: replib

Other DBs: None

Contributor: J. DEUTSCH,DEP. PHARMACEUT. CHEM.,HEBREW UNIV.,JERUSALEM,ISR

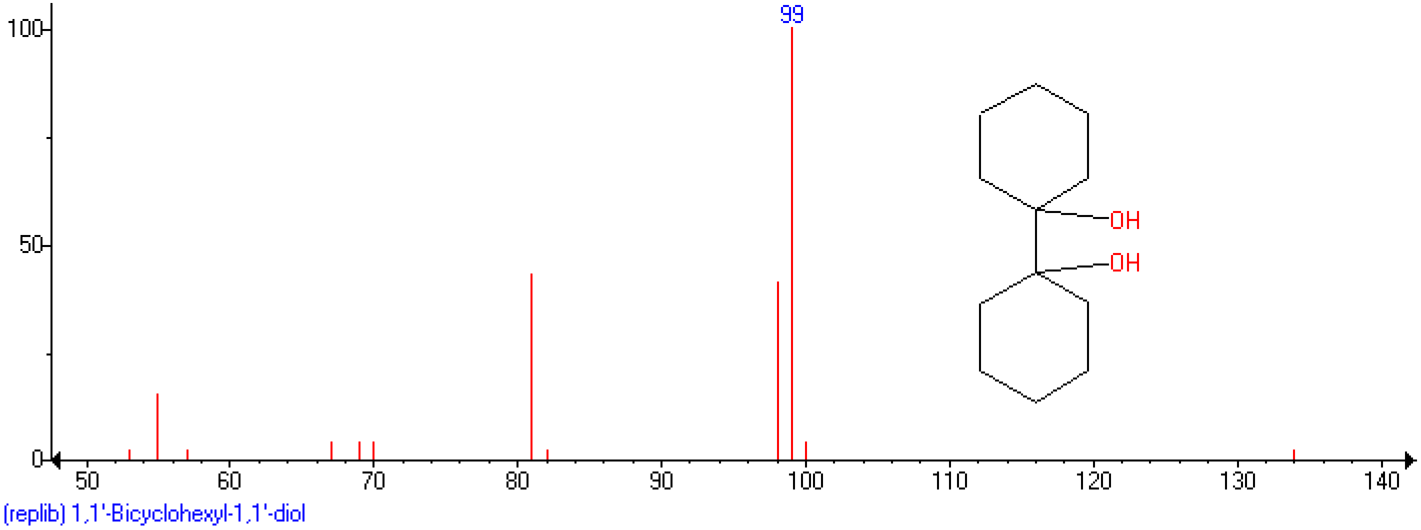

18.255

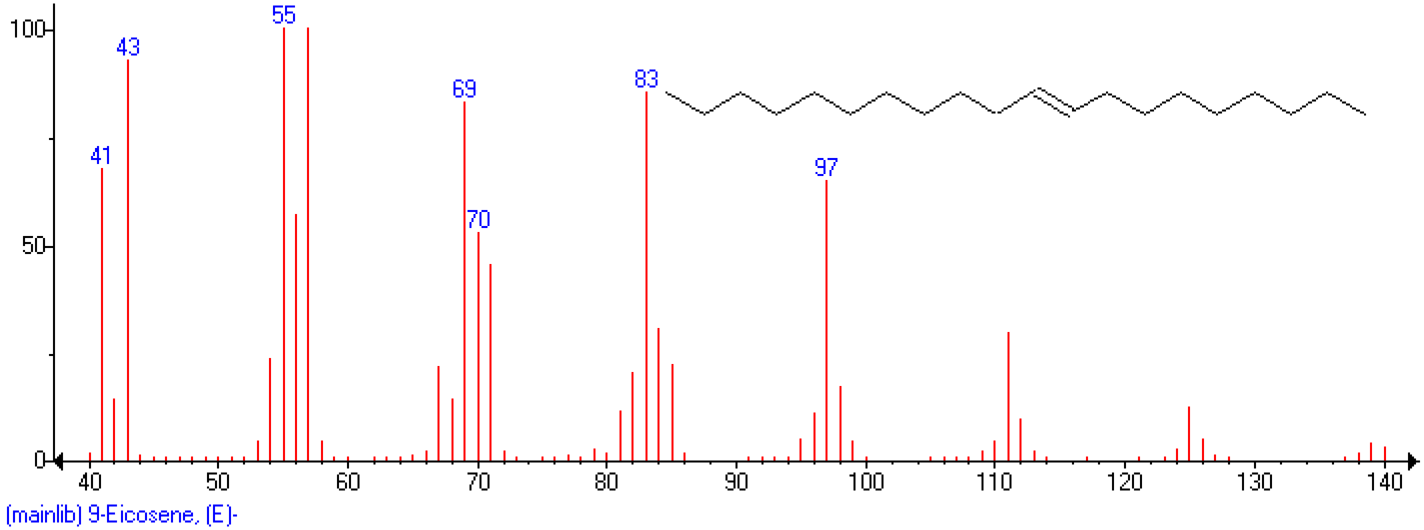

100
50
0
55
43
69
83
41
97
70
40
50
60
70
80
90
100
110
120
130
140
(mainlib) 9-Eicosene, (E)-
