# Supplementary material for: Microbial induced calcite precipitation can consolidate martian and lunar regolith simulants
Source: PLoS One. 2022 Apr 14;17(4):e0266415. doi: 10.1371/journal.pone.0266415 (PMC9009621; doi:10.1371/journal.pone.0266415)
Supplement: S1 Data — (ZIP) [file pone.0266415.s002.zip › Plos one_data file/raw_data_set/MSS_EDS.pdf]

New Project

Author: vandana
Creation: 07/07/2020 1:18:48 PM

Sample Name: New Sample

Area 111

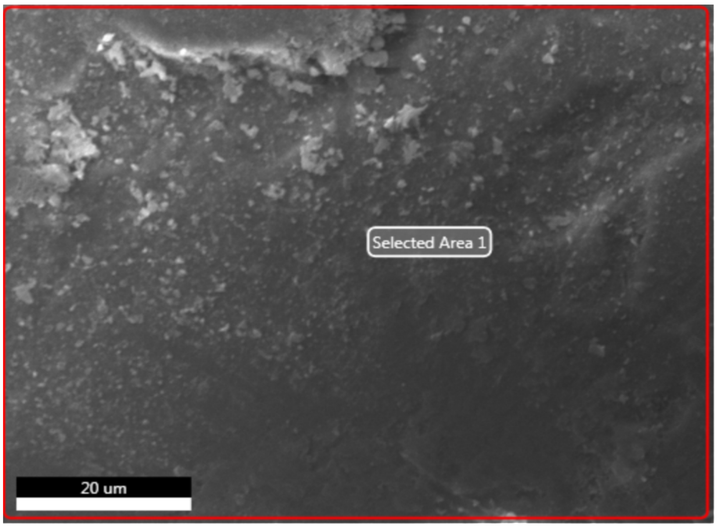

Notes:

Selected Area 1

kV: 20	Mag: 1500	Takeoff: 34.9	Live Time(s): 30	Amp Time(µs): 3.84	Resolution:(eV) 130.1

Selected Area 1 - Det 1

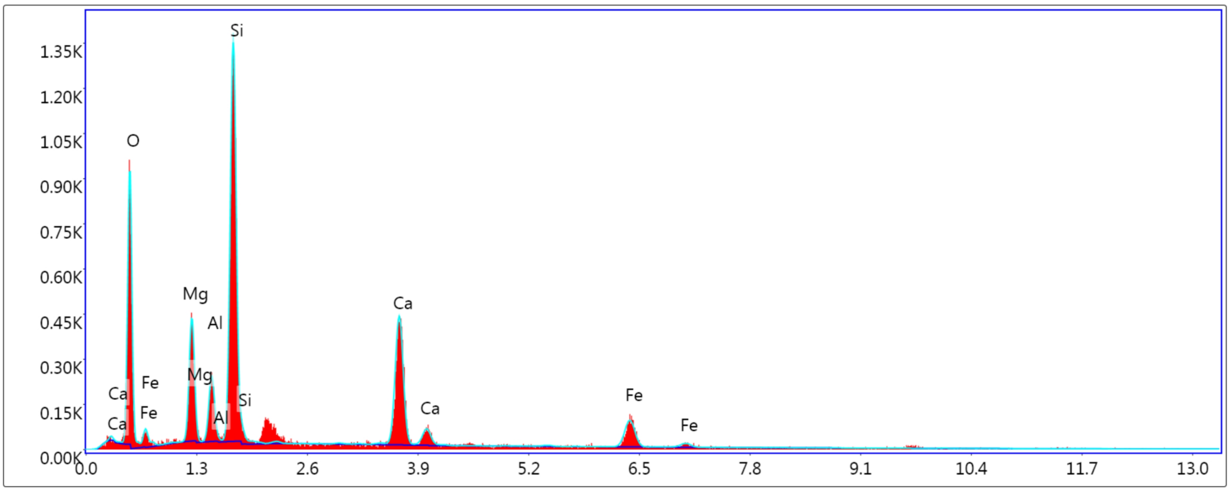

Lsec: 30.0 3 Cnts 9.820 keV Det: Element-C2B

eZAF Smart Quant Results

Element	Weight %	Atomic %	Net Int.	Error %	Kratio	Z	A	F
O K	41.05	57.96	195.95	10.25	0.1005	1.0706	0.2288	1.0000
MgK	8.64	8.03	108.25	8.31	0.0426	0.9913	0.4959	1.0028
AlK	4.31	3.61	59.76	9.59	0.0234	0.9550	0.5661	1.0045
SiK	23.37	18.80	389.38	5.53	0.1513	0.9763	0.6615	1.0027
CaK	15.41	8.68	168.26	4.03	0.1352	0.9230	0.9392	1.0125
FeK	7.23	2.92	44.00	8.42	0.0609	0.8239	0.9884	1.0345
