# Supplementary material for: Microbial induced calcite precipitation can consolidate martian and lunar regolith simulants
Source: PLoS One. 2022 Apr 14;17(4):e0266415. doi: 10.1371/journal.pone.0266415 (PMC9009621; doi:10.1371/journal.pone.0266415)
Supplement: S1 Data — (ZIP) [file pone.0266415.s002.zip › Plos one_data file/raw_data_set/raw LSS_EDS.pdf]

Comment: This report has been generated by Centre for Nano Science and Engineering (CeNSE), IISc. For the research work carried by Dr. Rashmi Dikshit

No peaks omitted

Processing option : All elements analyzed (Normalised)

Number of iterations = 4

Standard :

C CaCO3 1-Jun-1999 12:00 AM

O SiO2 1-Jun-1999 12:00 AM

Na Albite 1-Jun-1999 12:00 AM

Al Al2O3 1-Jun-1999 12:00 AM

Si SiO2 1-Jun-1999 12:00 AM

Ca Wollastonite 1-Jun-1999 12:00 AM

Element	Weight%	Atomic%
C K	6.02	9.47

Comment:

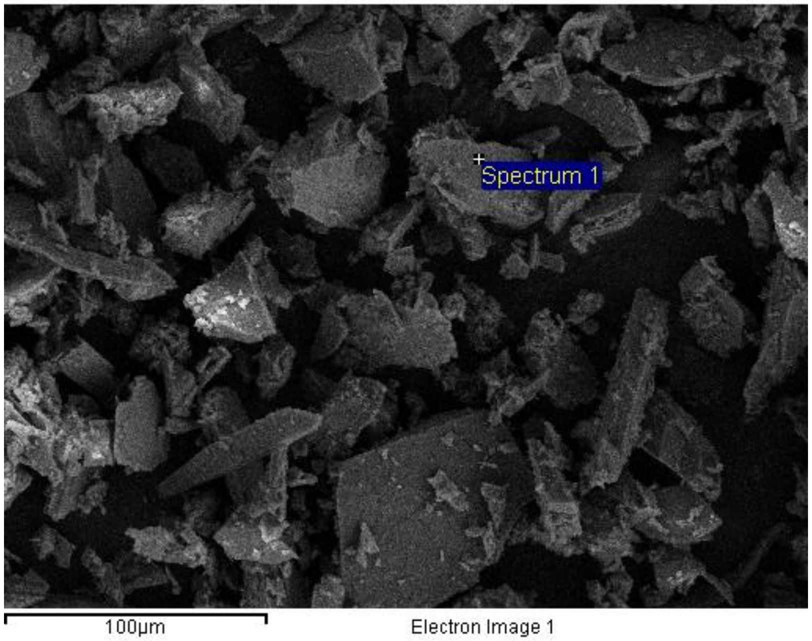

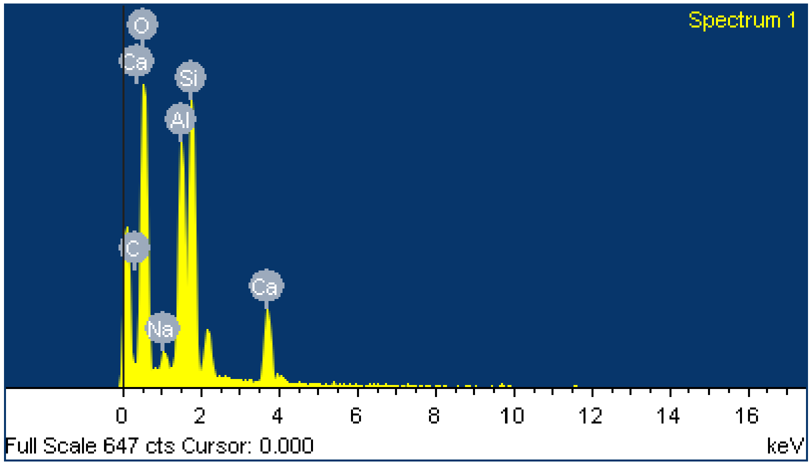

inca

O K	55.65	65.70
Na K	1.35	1.11
Al K	12.46	8.72
Si K	17.13	11.52
Ca K	7.39	3.48
Totals	100.00	

contol00GG

6/15/2019 2:10:38 AM

Spectrum processing :

No peaks omitted

Processing option : All elements analyzed (Normalised)

Number of iterations = 4

Standard :

O SiO2 1-Jun-1999 12:00 AM

Na Albite 1-Jun-1999 12:00 AM

Al Al2O3 1-Jun-1999 12:00 AM

Si SiO2 1-Jun-1999 12:00 AM

Ca Wollastonite 1-Jun-1999 12:00 AM

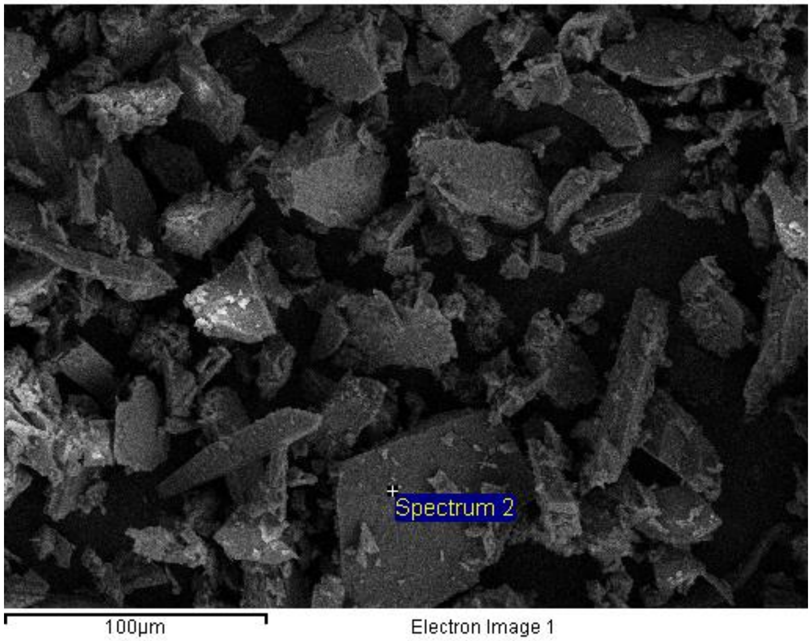

Element	Weight%	Atomic%
O K	51.16	65.75
Na K	1.29	1.15
Al K	16.34	12.45
Si K	21.14	15.48

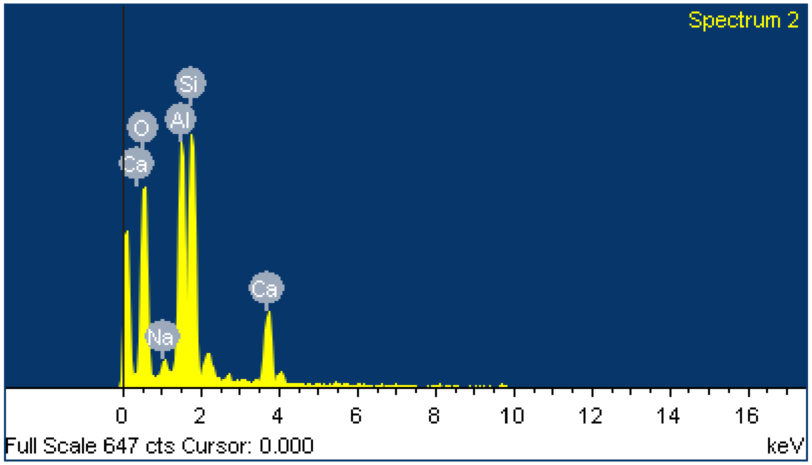

Comment:

Ca K	10.08	5.17
Totals	100.00	

contol00GG

6/15/2019 2:11:31 AM

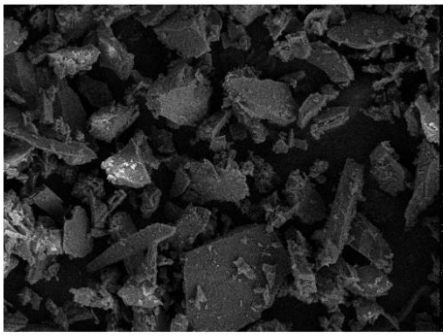

Electron Image 1

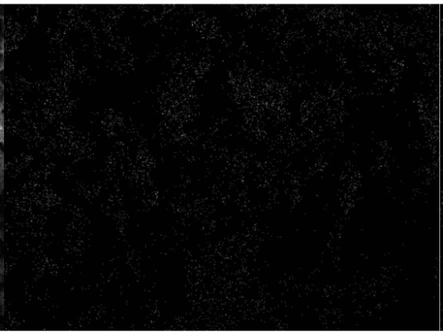

O Ka1

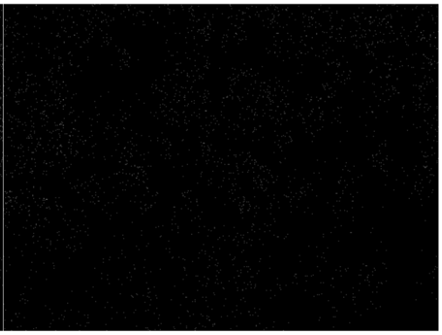

Ca Ka1

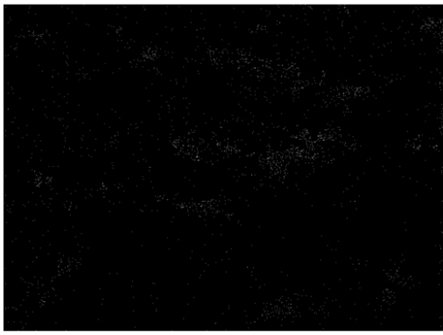

C Ka1_2

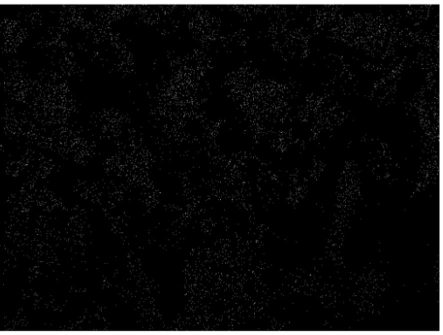

Si Ka1

Comment:
