# Supplementary material for: Microbial induced calcite precipitation can consolidate martian and lunar regolith simulants
Source: PLoS One. 2022 Apr 14;17(4):e0266415. doi: 10.1371/journal.pone.0266415 (PMC9009621; doi:10.1371/journal.pone.0266415)
Supplement: S1 Data — (ZIP) [file pone.0266415.s002.zip › Plos one_data file/raw_data_set/utm_data.pdf]

Compressive strength (MPa) for various treatments for MSS

Treatment	Compressive strength (MPa)	Std. Dev
MSS-SP-GG	1.18	0.30
MSS-SP-N	2.66	0.42
MSS-SP-GG-N	3.18	0.33

Compressive strength (MPa) for various treatments for LSS

Treatment	Compressive strength (MPa)	Std. Dev
LSS-SP	0.75	0.15
LSS-SP-GG	3.41	0.60
LSS-SP-N	1.63	0.37
LSS-SP-GG-N	5.65	0.26
