# Supplementary material for: Microbial induced calcite precipitation can consolidate martian and lunar regolith simulants
Source: PLoS One. 2022 Apr 14;17(4):e0266415. doi: 10.1371/journal.pone.0266415 (PMC9009621; doi:10.1371/journal.pone.0266415)
Supplement: Attachment — Submitted filename: response _reviewer_comments.docx [file pone.0266415.s004.docx]

**Response to Reviewer-1 comments**

**Manuscript ID**: PONE-D-21-30580**:**

**Manuscript title:** Microbial induced calcite precipitation can consolidate martian and lunar regolith simulants

This is an interesting idea and could lead to further developments. It is well written and clearly presented.

We would like to thank the referee for their generally positive comments on our manuscript. We present point-by-point clarifications to each of the comments below.

**Specific Comment # 1:** I do wonder why the authors used a Gram-positive bacterium, since a number of calcifying Gram negative bacteria have been investigated and have the ability to produce their own "gums" (extracellular polymeric substances, EPS), which could avoid the need to add yet another ingredient to the growth medium.

**Response**: We agree that there are numerous gram-negative strains reported for their potential for urease activity. These strains such as *Proteus mirabilis, Klebsiella aerogenes, Helicobacter pylori, Streptoccocus salivarius* and *Pseudomonas aeruginosa* are known to have encoded urease genes (ureA, ureB, and ureC) [1]. The main reason why we have not considered these strains is that they are pathogenic strains having enormous capability to cause various diseases in human beings. These microorganisms are known to colonize various organs like intestines, skin and airways etc., which favors the spread to other parts of the human body, especially in immunocompromised individuals [2].

Since our present work is largely motivated by the use of biocementation within the broader context of extra-terrestrial habitats, it is worthwhile to mention the Outer Space Treaty in this context, which emphasizes the use of space for peaceful purposes. The United Nations resolution adopted by General Assembly on 7^th^ December 2017 specifically called upon the Committee on the Peaceful Uses of Outer Space (COSPAR) to “promote the widest adherence to the Treaty, and its application by States, and to foster the progressive development of international space law”. In our discussions with scientists from both NASA (USA) and ISRO (India), the issue of bacterial pathogenicity has been an important focus; it has been felt that the use of pathogenic strains can be interpreted as an intent of non-peaceful use of space. Hence, keeping this issue in mind, we have deliberately chosen strains that are not pathogenic to human beings.

Furthermore, strains belonging to the *Bacillus* group are more commonly used for MICP applications as they are endospore producing strains having capability to tolerate extreme alkaline pH, dehydrating conditions and survive under high mechanical compressive forces etc. Sporulation provides an easy route for transport and packaging of bacteria for long duration flights where ambient conditions can change. Due to the above reasons, *Sporosarcina pasteurii*, which is an alkaliphilic, non-pathogenic, soil and endospore producing bacteria has been explored in the present study.

**Specific Comment # 2:** I should like the authors to include some comparisons with consolidated bricks produced by the other (non-microbial) methods that they mention. Are there advantages to this method and, if so, what are they?

**Response:** There are different methods that are being explored to produce consolidated mass through *in situ* construction material manufacturing methodologies such as sintering using laser beams [3], microwave irradiation [4] cast basalt production [5], lunar glass preparation [6], 3D printing [7] with different materials etc. Each method has its own sets of advantages and limitations. We now discuss some of these approaches in more detail below. These have also been mentioned in the revised manuscript in Introduction section.

The largest strength is produced, perhaps by the process of sintering. For instance, Gualtieri and Bandyopadhyay [8] prepared lunar regolith by mixing multiple simulants (JSC-1, JSC-1AF, and JSC-1AC) and consolidated them using cylindrical dies of 12.7 mm and 7 mm diameter, and a height to diameter ratio between 1.5:1 and 2:1. The samples were sintered at 1200 °C for 20 min and the resulting compressive strength was found to be between 103.2 to 232 MPa. Despite this significant strength, the sintering process is plagued by several inherent drawbacks such as high porosity, thermal cracking and difficulty in casting larger samples [9]. Also, clustering and coalescence of pores while sintering in microgravity makes the material less strong as compared to that sintered on earth [10]. In another work carried out by Young-Jae Kim *et al*. [13], KLS-1 lunar regolith simulant was consolidated with microwave sintering method at 1120 °C and compressive strength reported was approximately 39 MPa.

On the other hand, another common consolidation method - cast basalt production – has the problem of large shrinkage deformations in the resulting material [11], Besides the thermite reaction requires energy-intensive extraction, and sulfur concrete is not a universally viable solution due to large temperature variation on the moon [12].

In parallel efforts, some researchers have developed a photocurable lunar regolith simulant suspension to create small regolith bound structures which use the 3D vat polymerization (VP) method. These 3D printed structures achieved compressive strengths of approximately 5 MPa, comparable with the present study [7]. Finally, a very recent report has explored a biological route for making structural units of lunar and martian regolith using human serum albumin (HSA), a protein extracted from human blood plasma, as the binder These structures gave compressive strength of the order of 20 to 32 MPa [14] however the stability of the protein under general non-laboratory conditions remains a major challenge.

Other, less well-known methods for consolidation of regolith have also been pursued in the literature and are listed in Table R1.

Our approach is inspired by the consolidation processes that nature has long been using for building strong and sustainable habitats such as corals, spider webs, termite mounds, and anthills. We are proposing a casting method for fabrication of brick-like structures using regolith simulants via a biomineralization process called microbial induced calcite precipitation (MICP) in the present work. As a natural process, MICP is highly scalable, and the calcium carbonate nucleating inside the pore spaces can lead to materials with low porosity and significant strength.

This approach can also sequester environmental carbon dioxide into calcium carbonate and necessitates low temperature (30-40℃) to complete the process. Our consolidated bricks have a compressive strength equivalent to ice so they can, in principle, form the basis for building settlements in the future (not unlike igloos). Given the fact that the lunar gravity and martian gravity are ~1/6 and ~ 1/3 respectively of earth's gravity, this would correspond to a required strength of ~1.7–3.3 MPa and ~3.3-6.6 MPa respectively on those planets. These values are within the range of peak compressive stresses demonstrated in our present work.

.

**Table R1 (Review Document Only): Compressive strengths of lunar regolith simulants fabricated structure via different process**

| Adopted Method of consolidation | Material used | Obtained Compressive  strength | Reference of work |
| --- | --- | --- | --- |
| Binder jetting | Regolith, sorel cement,  binder liquid | 20 MPa | Cesaretti  et al. [15] |
| Extrusion | Regolith (72 wt%),  urea, alkaline solution | 13 MPa | Pilehvar  et al. [16] |
| Selective laser  Melting | Regolith | 4 MPa | Goulas et al.  [17] |
| Solar sintering | Regolith | 2 MPa | Meurisse  et al. [18] |
| Casting:  Geopolymer | Regolith (76 wt%),  liquid silicate, alkaline  solution | 16 MPa | Montes et al.  [19] |
| Casting: Sulphur  Concrete | Regolith (65 wt%),  Sulphur | 31 MPa | Toutanji  et al. [20] |
| Casting: Thermite  reaction | Regolith (67 wt%),  aluminium powder | 18 MPa | Faierson  et al. [21] |
| Extra-terrestrial regolith bio-composites with Human Serum Albumin and urea | Lunar and martian regolith | 20-32 | Roberts et .al [14] |

Data presented in the table has been adopted from Altan et .al. [7].

In light of the referee’s comment, the following new references have been added to the revised manuscript.

23. Gualtieri T, Bandyopadhyay A. Compressive deformation of porous lunar regolith. Mater Lett. 2015;143: 276–278.
26. Kim Y-J, Ryu BH, Jin H, Lee J, Shin H-S. Microstructural, mechanical, and thermal properties of microwave-sintered KLS-1 lunar regolith simulant. Ceram Int. 2021;47: 26891–26897.
27. Altun AA, Ertl F, Marechal M, Makaya A, Sgambati A, Schwentenwein M. Additive manufacturing of lunar regolith structures. Open Ceram. 2021;5: 100058.
28. Roberts AD, Whittall DR, Breitling R, Takano E, Blaker JJ, Hay S, et al. Blood, sweat, and tears: extraterrestrial regolith biocomposites with in vivo binders. Mater Today Bio. 2021;12: 100136.

**References cited in the response**

1.	Castro-Alonso MJ, Montañez-Hernandez LE, Sanchez-Muñoz MA, Macias Franco MR, Narayanasamy R, Balagurusamy N. Microbially induced calcium carbonate precipitation (MICP) and its potential in bioconcrete: microbiological and molecular concepts. Front Mater. 2019;6: 126.

2.	Oliveira J, Reygaert WC. Gram negative bacteria. 2019.

3.	Balla VK, Roberson LB, O’Connor GW, Trigwell S, Bose S, Bandyopadhyay A. First demonstration on direct laser fabrication of lunar regolith parts. Rapid Prototyp J. 2012.

4.	Cooper, B., Microwave sintering of regolith simulant. Space Resource News, 1992. 1(8): p. 1-3

5.	Greene KA. Design Note on Post-Tensioned Cast Basalt. Engineering, construction, and operations in challenging environments: Earth and space 2004. 2004. pp. 45–50.

6.	Allen CC, Hines JA, McKay DS, Morris R V. Sintering of lunar glass and basalt. Engineering, Construction, and Operations in space-III: Space’92. 1992. pp. 1209–1218.

7.	Altun AA, Ertl F, Marechal M, Makaya A, Sgambati A, Schwentenwein M. Additive manufacturing of lunar regolith structures. Open Ceram. 2021;5: 100058.

8.	Gualtieri T, Bandyopadhyay A. Compressive deformation of porous lunar regolith. Mater Lett. 2015;143: 276–278.

9.	Indyk SJ, Benaroya H. A structural assessment of unrefined sintered lunar regolith simulant. Acta Astronaut. 2017;140: 517–536.

10.	German RM. Gravitational Effects on Liquid Phase Sintering-Distortion Observations. Unpubl manuscript Retrieved from. 2003; 2003–2017.

11.	Happel JA. Indigenous materials for lunar construction. 1993.

12.	Carrier III WD. Particle size distribution of lunar soil. J Geotech Geoenvironmental Eng. 2003;129: 956–959.

13.	Kim Y-J, Ryu BH, Jin H, Lee J, Shin H-S. Microstructural, mechanical, and thermal properties of microwave-sintered KLS-1 lunar regolith simulant. Ceram Int. 2021;47: 26891–26897.

14. Roberts AD, Whittall DR, Breitling R, Takano E, Blaker JJ, Hay S, et al. Blood, sweat, and tears: extraterrestrial regolith biocomposites with in vivo binders. Mater Today Bio. 2021;12: 100136.

15.	Cesaretti G, Dini E, De Kestelier X, Colla V, Pambaguian L. Building components for an outpost on the Lunar soil by means of a novel 3D printing technology. Acta Astronaut. 2014;93: 430–450.

16.	Pilehvar S, Arnhof M, Pamies R, Valentini L, Kjøniksen A-L. Utilization of urea as an accessible superplasticizer on the moon for lunar geopolymer mixtures. J Clean Prod. 2020;247: 119177.

17.	Goulas A, Binner JGP, Engstrøm DS, Harris RA, Friel RJ. Mechanical behaviour of additively manufactured lunar regolith simulant components. Proc Inst Mech Eng Part L J Mater Des Appl. 2019;233: 1629–1644.

18.	Meurisse A, Makaya A, Willsch C, Sperl M. Solar 3D printing of lunar regolith. Acta Astronaut. 2018;152: 800–810.

19.	Montes C, Broussard K, Gongre M, Simicevic N, Mejia J, Tham J, et al. Evaluation of lunar regolith geopolymer binder as a radioactive shielding material for space exploration applications. Adv Sp Res. 2015;56: 1212–1221.

20.	Toutanji HA, Evans S, Grugel RN. Performance of lunar sulfur concrete in lunar environments. Constr Build Mater. 2012;29: 444–448.

21.	Faierson EJ, Logan K V, Stewart BK, Hunt MP. Demonstration of concept for fabrication of lunar physical assets utilizing lunar regolith simulant and a geothermite reaction. Acta Astronaut. 2010;67: 38–45.

**Response to Reviewer-2 comments**

**Manuscript ID**: PONE-D-21-30580**:**

**Manuscript title:** Microbial induced calcite precipitation can consolidate martian and lunar regolith simulants

I read with interest your paper about the microbial consolidation of regolith. I must say I expected some more-in-depth evaluations about the effect of carbonate precipitation on the mechanical properties of your material (maybe a grain size vs mechanical quality effect, or something related to the percentage of one calcium carbonate polymorph instead of the others, or the total amount of calcium carbonate precipitated comparing different conditions and so on). In fact, I didn't find what I expected. The paper does not enter into the details of the mechanisms triggered by the different preparative routes but simply compares some samples without trying an explication, a model, a generalization to forecast what will happen by changing one among the variables.

I put some comments directly into the text. Please check typos.

**Response**: We thank the reviewer for their comments and feedback on our initial manuscript. The primary challenge that we hoped to address in the present work is the great need for identifying microbial strains and culture conditions which can survive under extra-terrestrial conditions. This is a widely acknowledged challenge with regards to extra-terrestrial usage of the MICP process. We quote from a recent review article [Changela, HG., et al. "Mars: new insights and unresolved questions." International Journal of Astrobiology 20.6 (2021): 394-426.)] [1]. *One limitation may be the various salts that are known to inhibit or prevent microbial metabolism, including perchlorates and chaotropic salts, sulphate salts, and those that create multiple stresses and polyextreme conditions… Work is therefore needed to identify strains tolerant to salt-induced stresses and optimize culture conditions to mitigate against the underlying cellular stress mechanisms.*”

Our current work is specifically designed to address this challenge. While we believe one of the novelties of our work is in this demonstration by itself, we also understand the referee’s concern about developing a model to aid more detailed understanding of the bonding characteristics. In fact, we have already embarked on such a program involving a random spring network model [2]. Incorporation of these ideas into the present manuscript would necessitate the inclusion of large amounts of background material as basis for the random network model. This would deviate significantly from the scope of the present manuscript and has hence been avoided at this junction. However, we present some preliminary results below that directly help address the referee’s specific concerns.

A) Bond model for evaluating mechanical strength: We use a standard bond model [3] that treats each particle in the consolidated agglomerate as an individual node connected to adjacent nodes via suitably defined springs. One can think of the soil particles as constituting the nodes and the bridges between particles as being represented by the springs. The particles are ideally located on a lattice but nodes are removed with a probability equal to the desired porosity to simulate the effect of a disordered structure, typical of the consolidates in our experiments. Each spring extends/compresses in response to the local force at a particular node and breaks when the force reaches a critical value. In order to mirror the experiments, forces in the entire spring network are determined simultaneously due to externally applied compressive displacement at the boundaries. By incrementing these boundary displacements, we obtain a numerical load displacement curve, analogous to those measured in the compression test experiments.
B) Determination of model parameters: The three parameters in the model described above – structure porosity, individual bond stiffness and breaking force – are obtained by fitting the model prediction to those obtained experimentally. Porosity was measured using microCT (Bruker) and the mean bond stiffness and breaking strength were varied to fit the measured compressive loading curves, see Table R1 below.

**Table R1. Sample porosity and corresponding compressive strength as measured by 3D micro-CT reconstruction.**

| S. No | Sample Treatment | Compressive Strength (MPa) | Porosity (%) |
| --- | --- | --- | --- |
| 1 | LSS-SP | 0.75 | 50 |
| 2 | LSS-SP-N | 1.63 | 50 |
| 3 | LSS-SP-GG | 3.41 | 32 |
| 4 | LSS-SP-GG-N | 5.65 | 23 |

C) Bond strength distribution: In order to model uncertainties in the bond strengths (e.g., due to various polymorphs in the binder), we use a spatially varying bond strength to within +- 10% of the mean value obtained above (step B). This was then subject to the compressive loading simulation described in step A.

Sample realizations of the lattice at the end of these three steps are shown in Fig. R1 below

**Figure R1 (Response Document Only). (Left) Sample simulation realization with bonds coloured by bond stiffness. The colour bar is relative to the mean bond stiffness (here normalized to 1.0, variation +- 5%). The porosity of this particular sample was fixed at 20%. (Right) Gross sample compressive strength from simulations shows a sharp reduction with porosity. The error bar represents the error for data averaged over 5 different pore distribution realizations.**

Since the samples obtained from biocementation using the present process typically had porosity of between 20-30%, we also evaluated the effect of larger variations in bond stiffness (typical of polymorphs) on the resulting compressive strengths, the results are summarized in Fig. R2.

**Figure R2 (Response Document Only). Sample compressive strength variation for different uncertainty in individual spring stiffness, data for 20% and 30% porosity.**

We are preparing a separate manuscript to present these results along with a detailed discussion of both the model and its predictions.

In light of this, we wish to re-emphasize that the present manuscript is primarily focused on addressing the MICP process and its biological underpinnings, particularly in the context of extra-terrestrial soil simulants. Both soil simulants used in the present work have different chemical composition and hence we have focused on studying the effect of these on the performance of a particular bacterial strain of interest - *Sporosarcina pasteurii* (SP). As we’ve mentioned in section 3.2 of the manuscript, our initial results showed that some soil components from MSS were triggering inhibition of biological activity. This is the reason we embarked on exploring alternative pathways. Since the MICP process of SP is based on ureolytic activity by a metalloenzyme (urease) that has Ni at the centre, we supplemented the nutrient medium with Ni under flask condition, both with and without soil. Ammonium ion concentration was taken as the prime factor to evaluate biological activity as urease enzyme hydrolyses urea and releases ammonium ions. This favours the microenvironment by enhancing the media pH, thus accelerating the MICP process. Figure R3 shows the screening of temporal evolution of ammonium ion concentration with the supplementation of different concentration of nickel. Here we observe that after supplementation of 10 mM nickel chloride, there was a significant increase in the ammonium ion concentration in both cases. This figure has been incorporated in the supplementary file as figure S1.

**Fig. R3 (Included as Supplementary Fig. S1): Screening of temporal evolution of ammonium ion concentration with the supplementation of different concentration of Ni**

The concern raised by the reviewer regarding the total amount of calcium carbonate precipitated comparing different conditions is a welcome suggestion. To address this, one set of experiments was performed with different treatments to evaluate the total amount of calcium carbonate precipitated. Consolidated samples of both soil simulants under various conditions were first tested for compressive strength and subsequently processed for thermogravimetry analysis (TGA). The data revealed that guar gum and Ni supplementation together support the formation of crystalline phases of CaCO_3_ thereby facilitating the MICP process. The details are as described below. This text has also been included in the revised manuscript as follows.

Page # 7: In Sec. “MATERIALS and METHODS” under “Mechanical and Materials Characterizations”

*For* *thermogravimetry (TG) (Mettler Toledo TGA analyser STARe SW10.00,) analysis of space bricks, ~10 mg soil samples were taken from each treatment separately and placed on a cylindrical 70 μL alumina crucible followed by heating from 0 °C to 1000 °C. The heating rate was set at 10 °C/min throughout the experiment*

Page # 14: In Sec. “RESULTS AND DISCUSSION” under ‘TG characterization of space bricks’

*Thermal analysis for the precipitates was performed for the consolidated space bricks as shown in Fig. 7b, c and d (for MSS) and Fig. 8b, c and d (for LSS) consolidated bricks, respectively. The TG curves can be divided into three regions based on the transitions observed due to thermal decomposition. The first part of the curve within the range of 50 to 250℃ can be attributed to the loosely bound water or moisture content present in the samples* [57]. *The second part in the temperature range of 300 to 800℃ can further be divided into two stages of decarbonation. The first stage is between 300 to 500℃ where decomposition of poorly crystalline phase of CaCO_3_ occurs and in the second stage (500 to 800℃), decomposition of vaterite and calcite occurs* [58,59]. *It can be observed from Fig 7c and 8c that the supplementation of Ni reduces the decomposition of the first poorly crystalline phase. It appears that Ni supplementation (Ni being a central part of the urease enzyme) accelerates the formation of crystalline phases of CaCO_3_ in both the soil simulants as compared to guar gum supplementation alone. Maximum decomposition with both soil simulants was observed in the range of 500 to 700℃ (around 3.2% in case of LSS-SP-N and 3% in case of MSS-SP-N) which supports the formation of calcite and vaterite phases. However, this crystalline phase was further extended in the treatment supplemented with Ni and guar gum together with both the soil simulants (around 5% in case of LSS-SP-GG-N and about 4.2% in case of MSS-SP-GG-N), see Fig. 7d and 8d. Based on these thermal decomposition results, it can be concluded that guar gum and Ni supplementation together support the formation of vaterite and calcite, thereby augmenting the MICP process.*

**Fig. 7a) XRD spectrum of consolidated martian bricks with different treatments. (V – vaterite; C- calcite; A- aragonite). TG curves: b) MSS supplemented with guar gum and treated with *Sporosarcina pasteurii* c) MSS supplemented with Ni and treated with *Sporosarcina pasteurii* d) MSS supplemented with guar gum and Ni and treated with *Sporosarcina pasteurii.***

**Fig. 8a) XRD spectrum of consolidated lunar bricks with different treatments. (V – vaterite; C- calcite; A- aragonite). TG curves: b) LSS supplemented with guar gum and treated with *Sporosarcina pasteurii* c) LSS supplemented with Ni and treated with *Sporosarcina pasteurii* d) LSS supplemented with guar gum and Ni and treated with *Sporosarcina pasteurii.***

**Specific Comment # 1:** Section 1 last para, what does guar gum stabilizes? It is used like a glue, or does it work as a chemical binder?

**Response**: Guar gum is a non-ionic, galactomannan polysaccharide which forms a highly structured cohesive gel and has the ability to form hydrogen bonds [4]. Guar gum has been studied as a binder material by several groups and the following hypotheses have been proposed for its binding action [4, 5]. Firstly, guar gum has electrokinetic charges that ensure formation of strong bonds with soil particles, which is further supplemented by the presence of borate ions. Secondly, the presence of hydroxyl groups in guar gum enables it to form additional hydrogen bonds with soil particles as well as in the solution [4]. Hence it appears likely that similar mechanisms are at work in our experiments as well. However, the precise binding mechanisms with soil and bio-cementation continues to be a topic of active investigation.

Furthermore, guar gum has the added advantage of extending the lifespan of bacteria, see Fig. R4. Panel (a) in this figure shows bacteria along with the precipitates after 5 days of incubation and panel (b) presents powder XRD results that clearly indicate the occurrence of various phases of calcium carbonate, just as discussed earlier.

**Fig. R4 (Response Document Only). Micro-structural characterization of *S. pasteurii* induced precipitates inoculated in medium supplemented with guar gum (1% w/v) a) Scanning electron microscopic view of precipitates b) PXRD pattern of precipitates. In the spectrum, V represents vaterite and C calcite. Incubation period was 5 days in all cases.**

Finally, preliminary evidence shows that addition of guar gum also leads to the production of auxiliary compounds that could have led to enhanced bacterial activity (See Fig.9a & b). Data pertaining to GCMS profiling of metabolites for treatments with and without guar gum supplementation has been incorporated in the revised manuscript.

Page # 6: In Sec. “MATERIALS and METHODS” under “Microorganisms and culture conditions” following text have been incorporated.

*Gas Chromatography Mass Spectrometry (GCMS) profiling was performed for metabolites mapping produced with and without guar gum supplementation in the SM medium. For GCMS (GC column: Agilent 7890A MS: 5975C MSD Electron Impact Ionization Mass Analyze equipped with Quadrupole Software), 1 μL of methanol extract of bacterial metabolites was injected into the GC inlet. Mass spectral library (NIST2011) was used to identify the fraction based on their mass spectra (m/z ratio).*

Page # 16: We have added the following text in “RESULTS AND DISCUSSION’ section.

*Guar gum has been studied as a binder material by several groups and the following hypotheses have been proposed for its binding action [60, 61]. Firstly, guar gum has electrokinetic charges that ensure formation of strong bonds with soil particles, which is further supplemented by the presence of borate ions. Secondly, the presence of hydroxyl groups in guar gum enables it to form additional hydrogen bonds with soil particles as well as in the solution [60]. Hence it appears likely that similar mechanisms are at work in our experiments as well. However, the precise binding mechanisms with soil and bio-cementation continues to be a topic of active investigation.*

*In addition, bacterial activity relevant to the MICP process due to guar gum supplementation was comprehended by GCMS profiling of secondary metabolites. The metabolites in both treatments (SM and SM-GG) were segregated into aliphatic, aromatic and nitrogenous compound, as shown in fig. 9a and the names of the identified metabolites are listed in fig 9b. In SM-GG treatment, more fused ring aromatic compounds such as methylbenzoyl) amino] (phenylamino)methylene]-, S-methyl ester d, Isophthalic acid, ethyl 4-formylphenyl ester were observed as compared to SM treatment. These additional macromolecules observed in SM-GG treatment could be one of the reasons for increased bacterial metabolic activity leading to enhanced calcium carbonate precipitation and increased strength of space brick. Some important nitrogenous compounds such as 2-Dimethylamino-3-methylpyridine, 4-Fluorohistamine, and Picolinamide were also observed exclusively with SM-GG treatment. There were few common aromatic compounds like Naphthalene, Benzo[b]thiophene, 1-Dodecene, Tridecane and Carbamodithioic acid recorded in both the treatments. One organic compound namely Carbamic acid, N-(2,3-dimethylphenyl)-, oxiranylmethyl ester was observed only in guar gum supplemented medium. Carbamic acid and its derivatives are unstable compounds. Carbamic acid is produced as an intermediate compound in urea hydrolysis reaction. It is reported that organic macromolecules can facilitate the biomineralization process either by providing a structural framework, or regulating its dynamic process such as the nucleation site, orientation and growth of crystal [62–64] etc. as observed with the SP-GG treatment. This constitutes an active area of investigation and has important consequences for the binding mechanics of guar gum.*

**Fig 9 a): GCMS profiling for different combination of growth media namely SM without any media additives, SP-GG supplemented with guar gum, inoculated with *Sporosarcina pasteurii* under flask condition after 6 days of incubation. SM represent for the treatment of SM without supplementation of guar gum whereas GG for the guar gum supplementation. Hatched column representing common compounds in both treatments. b) Showing name of the metabolites obtained from GCMS.**

The following new references have been incorporated in the revised manuscript

57. Brittain HG. Profiles of drug substances, excipients, and related methodology. Academic press; 2020.
58. Liu M, Cai L, Luo H. Effect of nano-silica on microbiologically induced calcium carbonate precipitation. Constr Build Mater. 2022;314: 125661.
59. Wang D, Chang J. Comparison on accelerated carbonation of β-C2S, Ca (OH) 2, and C4AF: Reaction degree, multi-properties, and products. Constr Build Mater. 2019;224: 336–347.
60. Sujatha ER, Saisree S. Geotechnical behaviour of guar gum-treated soil. Soils Found. 2019;59: 2155–2166.
61. Chang I, Im J, Prasidhi AK, Cho G-C. Effects of Xanthan gum biopolymer on soil strengthening. Constr Build Mater. 2015;74: 65–72.
62. Mann S. Biomineralization: principles and concepts in bioinorganic materials chemistry. Oxford University Press on Demand; 2001.
63. Polowczyk I, Bastrzyk A, Fiedot M. Protein-mediated precipitation of calcium carbonate. Materials (Basel). 2016;9: 944.
64. Nawarathna THK, Nakashima K, Fujita M, Takatsu M, Kawasaki S. Effects of cationic polypeptide on CaCO3 crystallization and sand solidification by microbial-induced carbonate precipitation. ACS Sustain Chem Eng. 2018;6: 10315–10322

**Specific Comment # 2:** Section 1 last line, “We believe that such studies will help evaluate
the possibilities of MICP as a sustained solution for building extra-terrestrial settlements
utilizing *in situ* resources.” How do you think to adapt the miniaturized environment to buildings?

**Response**: We believe that the work presented in this manuscript, and indeed our on-going work on space bricks themselves, constitute the first tentative steps towards realization of large-scale extra-terrestrial settlements. For context, the National Aeronautics and Space Administration (NASA) estimates that the cost of shipping one pound of construction material to outer space is of the order of 10,000 USD [6]. The obvious financial implications notwithstanding, this is also not a practical solution so that any potential habitat development must rely on the use of *in situ* resources. The key metric in evaluating the feasibility of this process is in the compressive strength of the resulting consolidates. In this regard, we note that the compressive strength of clay bricks used for general construction purposes on earth (g = 9.81 m/s2) is around 10–20 MPa. The primary loading in these cases is due to the structure’s self-weight. Given that the lunar and martian gravity are ~1/6 and ~ 1/3 respectively of that on earth, an equivalent strength of ~1.7–3.3 MPa and ~3.3-6.6 MPa would be required, respectively. Our laboratory scale experiments certainly show that this is clearly attainable using just the MICP driven bio-cementation process. We hope that this will spur more intense research on scaling up this process for large scale *in situ* resource utilization.

**Specific Comment # 3:** Section 3.1. Particle size characterization. Are these particles composed by MICP + Regolith powder?

**Response:** The particle size data presented in Sec. 2.2 was obtained for virgin soil simulants prior to any form of consolidation. This has been reemphasized in the revised manuscript (see Sec. 2.2 “Characterization of unconsolidated (raw) soil simulants”).

**Specific Comment # 4:** Section 3.1 XRD of raw soil samples. A) Chemical composition of MSS is related to the regolith composition. This is something that must be known in advance to choose right precursor. B) “It is reported that higher concentrations of iron may lead to toxic effects on bacterial cells due to generation of reactive oxygen compounds”. This is something to be considered in ad values, before using it as the precursor.

**Response**: As the referee suggests, the composition of the extra-terrestrial soil simulants is available. However, we must note that since the soil simulants are a very niche product that are provided either by nationalised space agencies (such as ISRO) or very specialized labs such as University of Central Florida’s Exolith Lab. As a non-commercial, batch produced, product, slight variations from sample to sample are expected and have even been observed. Hence, at our lab all samples are separately tested and their data matched with manufacturer’s own datasheets to ensure reproducibility and data availability. Thus, to ensure that readers have available to themselves all relevant data now and in the future, we decided to keep the XRD related to the soil.

As we’ve mentioned earlier, our basic intent in this work was to use a well-established bacterial strain (SP) which has been studied widely in the context of MICP. Further, we were also aware that urease (for the uerolytic pathway) from SP is a metalloenzyme containing Ni at the centre. Hence, we reasoned that Ni ion supplementation at an optimised concentration could accelerate the overall MICP process by enhancing urease activity. When we first attempted this, the preliminary results were very encouraging and this eventually led to the present study. Additionally, we wanted a common solution for both the soil samples (with composition shown in table R2) so that a standardized process can be established for obtaining bricks of significant compressive strength from different extra-terrestrial soil samples.

While attempting such a solution, we are also acutely aware of potential limitations that may arise due to various salts that are known to inhibit microbial metabolism. These include perchlorates and iron, sulphate salts, and those that create multiple stresses and polyextreme conditions [1 & 7]. Our group is working on addressing these aspects for identification of strains that are tolerant to salt induced stresses and also to optimize culture conditions to mitigate these concerns. We have isolated a *Bacillus* strain namely *Bacillus velezensis* (BV) that genetically shows tolerance against iron and sulphate ions. We have proceeded with the consolidation of the MSS and achieved encouraging results as shown in figure below (see Fig, R6). We hope to present a comprehensive description of these results in a forthcoming manuscript.

**Fig. R6 (Response Document Only) a) Compressive strengths of martian bricks mediated by** ***Bacillus velezensis* b) showing SEM micrograph of martian bricks**

**Table R2 (Response Document Only): Chemical composition of soils used**

| **Chemical Composition** | **Martian soil simulant (MSS)** | **Lunar soil simulant (LSS)** |
| --- | --- | --- |
| SiO_2_ | 48.3 | 39.79 |
| Al_2_O_3_ | 9.5 | 35.48 |
| Fe_2_O_3_ | - | 1.76 |
| TiO_2_ | 0.2 | 0.17 |
| CaO | 6.7 | 17.24 |
| MgO | 12.1 | 1.2 |
| Na_2_O | 2.6 | 3.09 |
| K_2_O | 0.1 | 0.03 |
| FeO | 6.9 | - |
| Cr_2_O | 0.1 | - |
| MnO | 0.1 | - |
| P_2_O_5_ | 0.2 | - |
| SO_3_ | 3.2 | - |

**Specific Comment # 5:** Before the experiment that Mg and Fe may inhibit urease activity, so why you chose that precursor for MSS.

**Response**: As mentioned in our response to the previous comment, our main focus in this work has been to seek a common solution for both soil simulants. Further, our isolated organism with optimised conditions has shown encouraging results with MSS containing Mg and Fe ions.

**Specific Comment # 6:** Section 3.3, paragraph 3. I must highlight that it is interesting that calcite in LSS sample shows will defined (104) signal as compared with MSS (104) is the most important crystal plane in calcite (more than 80% of the energy of the whole is contained in the (104) plane. If you cannot detect its signal in the XRD pattern of the MSS sample, this must be investigated.

**Response**: This is certainly an astute observation by the referee. In light of this comment, we revisited the original figure. We submit that the peak corresponding to (104) plane in the calcite samples was missed while marking them in the XRD graphs since it had a rather low relative intensity (<10%), see Table R3. We have nonetheless revised this figure and mentioned these details in the revised manuscript.

Table R3: Detail data of 2θ, d spacing, identified phase, hkl and corresponding ICSD references for the martian soil simulants

| **Treatment** | **2θ (Degree)** | **d spacing**  **(Å)** | **Phase** | **hkl** | **ICSD Ref.** | **Relative Intensity (%)** |
| --- | --- | --- | --- | --- | --- | --- |
| **MSS-SP-GG-N** | 29.2 | 3.05 | C | 104 | 98-002-3930 | 12.7 |
| **MSS-SP-GG** | 29.5 | 3.02 | C | 104 | 98-000-5337 | 6.9 |
| **MSS-SP-N** | 29.3 | 3.03 | C | 104 | 98-000-5339 | 8.3 |

**Specific Comment # 7:** section 3.4 Paragraph 1. As we added NiCl_2_. What about the effects of the counter ion (Cl^-^ or the ionic strength? Do you tested some other chlorides (i.e., CaCl_2_) to keep the ionic strength Cl^-^ content compared the results?

**Response:** Bacterial growth medium by itself contains various compounds (Table R4) which can indeed produce counter ions. However, considering only the Cl^-^ ions, the supplementation of amount of counter Cl^-^ ions produced by NaCl is larger compared to that produced by NiCl_2_. In the present study, we have not encountered any adverse effects on the MICP process due to the presence of counter ions. However, the possible effects of counter ions produced by the supplementation of other Ni based compounds such as nickel phosphate and nickel sulphate has not been explored in the current study.

**Table R4: Molarity of media composition used**

| **Constituent** | **Molarity used** |
| --- | --- |
| NaCl | 0.086M |
| NiCl_2_ | 0.01M |
| KH_2_PO_4_ | 0.015M |
| C_6_H_10_CaO_6_ | 0.05M |
| Yeast Extract (containing various amino acids, dextran, trehalose etc. | 2g/100g |

**Specific Comment # 8:** what is E.C. 3.5.1.5 ?

**Response:** This is the enzyme classification number to characterize the enzyme based on the reactions they catalyse. This number is generated in this format based on the recommendations of the Nomenclature Committee of the International Union of Biochemistry and Molecular Biology on the Nomenclature and Classification of Enzymes.

**Specific Comment # 9:** Two different instruments were used for XRD analysis?

**Response:** We have used only PANalytical Philips diffractometer to analyse our data. This correction is mentioned in the revised manuscript.

**References cited in the response**

1. Changela HG et al 2021. Mars: new insights and unresolved questions. International Journal of Astrobiology 20, 394–426.
2. Alava, M.J., Nukala, P.K. and Zapperi, S., 2006. Statistical models of fracture. Advances in Physics, 55(3-4), pp.349-476.
3. Herrmann, H.J. and Roux, S. eds., 2014. Statistical models for the fracture of disordered media. Elsevier.
4. Evangelin Ramani Sujatha, Sivaraman Saisree., 2019. Geotechnical behaviour of guar gum-treated soil. Soils and Foundations. 59 . pp.2155–2166.
5. Chang I, Im J, Prasidhi AK, Cho G-C. Effects of Xanthan gum biopolymer on soil strengthening. Constr Build Mater. 2015;74: 65–72.
6. E.J. Faierson, K.V. Logan, Potential ISRU of lunar regolith for planetary habitation applications, Moon, Springer, 2012, pp. 201–234.
7. Benison KC, O’Neill WK, Blain D and Hallsworth JE2021 Water activities of acid brine lakes approach the limit for life. Astrobiology 21: pp.729–740.
